# Urinary thromboxane and isoprostane levels are elevated in symptom-high T2-biomarker-low severe asthma

**DOI:** 10.1183/23120541.01089-2024

**Published:** 2025-08-26

**Authors:** Matthew Chad Eastwood, John Busby, Johan Kolmert, Javier Zurita, Sven-Erik Dahlén, Pamela Jane McDowell, Judy Bradley, David Jackson, Ian Pavord, Ratko Djukanovic, Joseph Arron, Peter Bradding, Chris Brightling, Rekha Chaudhuri, Douglas Cowan, Stephen Fowler, Timothy Colin Hardman, Cecile Holweg, James Lordan, Adel Mansur, Douglas Robinson, Craig E. Wheelock, Liam Heaney, on behalf of the investigators for the UK MRC Refractory Asthma Stratification Programme

**Affiliations:** 1 Centre for Experimental Medicine, School of Medicine, Dentistry and Biomedical Sciences, Queen's University, Belfast, UK; 2 Unit of Integrative Metabolomics, Institute of Environmental Medicine, Karolinska Institutet, Stockholm, Sweden; 3 Department of Respiratory Medicine, Karolinska University Hospital, Stockholm, Sweden; 4 Guy's and St Thomas’ NHS Foundation Trust, London, UK; 5 Oxford Respiratory NIHR BRC, Nuffield Department of Medicine, The University of Oxford, Oxford, UK; 6 University of Southampton, NIHR Southampton Biomedical Research Centre, Southampton, UK; 7 Sonoma Biotherapeutics, South San Francisco, CA, USA; 8 Department of Infection, Immunity and Inflammation, Institute for Lung Health, University of Leicester, Leicester, UK; 9 NHS Greater Glasgow and Clyde Health Board, Gartnavel Hospital, Glasgow, UK; 10 NHS Greater Glasgow and Clyde, Stobhill Hospital, Glasgow, UK; 11 Division of Infection, Immunity and Respiratory Medicine, University of Manchester, Manchester, UK; 12 Niche Science and Technology Ltd, Richmond, UK; 13 Genentech Inc., South San Francisco, CA, USA; 14 AbbVie, Mettawa, IL, USA; 15 The Newcastle upon Tyne NHS Foundation Trust, Newcastle upon Tyne, UK; 16 University Hospitals Birmingham NHS Foundation Trust, Birmingham, UK; 17 University College Hospitals NHS Foundation Trust, London, UK; 18 Unit of Integrative Metabolomics, Institute of Environmental Medicine, Karolinska Institute, Stockholm, Sweden

## Abstract

**Background:**

∼5–10% of patients with asthma have severe disease. A proportion remain symptomatic despite suppression of T2-related inflammation but what drives persistent symptoms remains unclear. Eicosanoids exert a functional role in pulmonary inflammation. We explored the relationship between urinary eicosanoids, asthma symptoms, obesity and T2-biomarker status.

**Methods:**

Urine was sampled during a randomised controlled trial assessing corticosteroid optimisation using T2-biomarker directed care at scheduled study visits (n=728) and at exacerbation (n=103). Urine eicosanoid concentrations were measured by mass spectrometry, then log2-transformed, z-scored and concatenated by biosynthetic pathway generating six pathway scores. Results were stratified by T2 status (T2-low: exhaled nitric oxide fraction (*F*_ENO_) <20 ppb and blood eosinophil count (BEC) <0.15×10^9^ cells·L^−1^; *versus* T2-high: *F*_ENO_ ≥20 ppb and BEC ≥0.15×10^9^ cells·L^−1^), symptoms (symptom-low: Asthma Control Questionnaire-7 (ACQ-7) <1.5; *versus* symptom-high: ACQ-7 ≥1.5) and obesity.

**Results:**

Isoprostane (pathway score p=0.02) and thromboxane (pathway score p=0.04) levels were higher in symptom-high *versus* symptom-low, T2-low participants. Isoprostane levels were greater in symptom-high *versus* symptom-low participants, irrespective of T2 status (pathway score p=0.01). Cysteinyl-leukotriene E_4_ levels (LTE_4_) were elevated in T2-high *versus* T2-low participants (pathway score p=0.0007), irrespective of symptoms. Corticosteroid exposure, obesity and exacerbations were not associated with increased eicosanoid levels (p≥0.05).

**Conclusion:**

Raised urinary eicosanoid levels of isoprostanes and thromboxanes were associated with increased symptoms in T2-low severe asthma. Elevated excretion of these metabolites in T2-low participants could reflect increased thromboxane-receptor (TP) activation, which may be promoting increased asthma severity and bronchoconstriction. Further research and interventions are needed to explore the role of TP modulation in T2-low severe asthma.

## Shareable abstract

**In T2-biomarker-low severe asthma, participants with high symptom scores displayed increased urinary eicosanoid levels of thromboxanes and isoprostanes. These metabolites are potent bronchoconstrictors that can act on the thromboxane receptor.** https://bit.ly/4hhK1ZB

## Introduction

∼5–10% of patients with asthma have severe disease with the majority exhibiting the “eosinophilic phenotype” [1, 2]. Despite suppression of T2-mediated eosinophilic inflammation (T2-high: exhaled nitric oxide fraction (*F*_ENO_)>25 ppb and blood eosinophil count (BEC) *>*0.15×10^9^ cells·L^−1^) a proportion of patients remain symptomatic [2, 3]. Previous studies describe a group of predominantly female patients, often T2-low, characterised by obesity (body mass index: (BMI) ≥30 kg·m^−2^) and frequently overtreated with corticosteroid (CS) therapies due to increased symptoms [3, 4]. It remains unclear what drives persistent symptoms in this group; however, it is hypothesised that obesity may contribute [3].

Eicosanoids are bioactive lipid mediators produced through the enzymatic and/or nonenzymatic oxidation of arachidonic acid *via* the cyclooxygenase (COX), lipoxygenase (LOX) and cytochrome-P450 (CYP450) pathways as well as free radical-induced peroxidation (leading to isoprostane formation) [5–8]. These pathways play important roles in maintaining normal physiological function and inflammatory cell signalling with mast cells, innate lymphoid cells and eosinophils implicated as mediators of inflammation in multiple diseases, including asthma [5, 8]. The end-scale metabolites of these pathways can be measured in urine [9, 10]. Within the respiratory milieu, eicosanoids can cause bronchoconstriction/relaxation (*via* interaction with thromboxane (TP), prostaglandin-D, prostaglandin-E, prostaglandin-F and cysteinyl-leukotriene (CysLT) receptors), resulting in inflammation by chemotactic signalling, mast cell activation and the release of T2-cytokines [5, 10, 11]. Whilst CysLTs, thromboxane-A_2_ (TXA_2_) and prostaglandin-D_2_ (PGD_2_) exhibit potent proinflammatory effects, primary prostaglandin-E_2_ (PGE_2_) is considered broncho-protective (anti-inflammatory) in human small airways and is proposed to stabilise mast cells [12]. Previous work found that eicosanoid concentrations were not affected by CS exposure [13]. Subsequently, eicosanoid profiling has been proposed as a noninvasive method to characterise asthma severity and identify patients exhibiting a T2-phenotype [9, 13].

A recent randomised control trial (RCT) compared CS treatment adjustment using a composite T2-biomarker score (*F*_ENO_, BEC, serum periostin) to a symptom-based scoring algorithm (symptoms, lung function, exacerbation history) in participants with severe asthma [14]. During this study, urine samples were collected at scheduled and exacerbation visits enabling the possibility to explore eicosanoid profiles in T2-low severe asthma. We hypothesised that elevated eicosanoids may contribute to persistent symptoms in T2-low severe asthma. We explored the stability of eicosanoid levels across scheduled study visits and examined the relationship between eicosanoids, symptoms, T2-biomarkers and obesity.

## Methods

### Study design and participants

**FIGURE 1:**
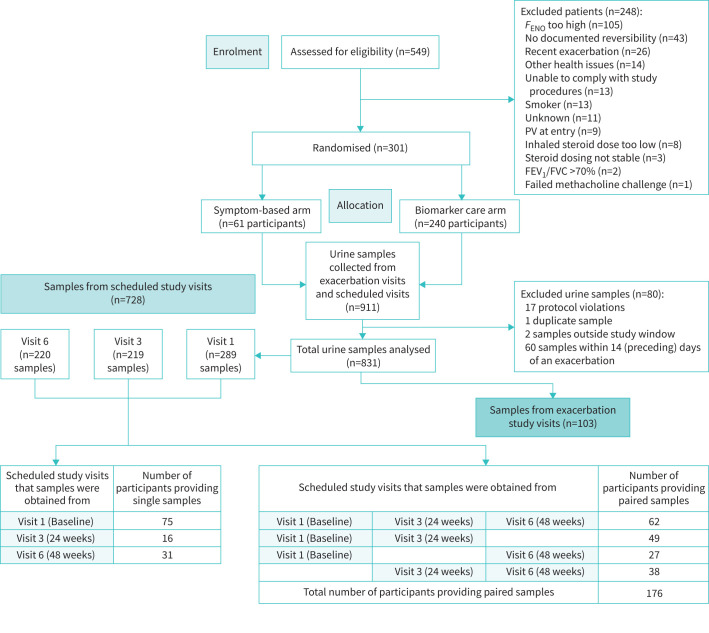
Consort diagram for study showing time points at which urine samples were taken during scheduled study visits and exacerbation visits, for analysis of urinary eicosanoids. *F*_ENO_: fractional exhaled nitric oxide; FEV_1_: forced expiratory volume in 1 s; FVC: forced vital capacity; PV: protocol violation.

This single-blinded (study participant), multicentre, 48-week RCT recruited participants with severe asthma (Global Initiative for Asthma, steps 4 and 5) [1, 15]. A composite T2-biomarker score (Biomarker-directed care) was compared to a symptom-based algorithm (Standard care) to facilitate CS treatment optimisation in participants with severe asthma [14]. Participants (n=301), aged 18–80 years from 12 UK specialist severe asthma centres, were randomised, following consent, in a 4:1 ratio to a biomarker-directed care or standard-care arm (figure 1). This study was enriched for T2-low severe asthma participants at screening (for full inclusion and exclusion criteria, see supplementary material) [1].

The trial design, study protocol (Clinicaltrials.gov NCT02717689) and primary study outcome have been published [14, 15]. The primary study protocol was approved by the Office for Research Ethics Committees Northern Ireland (NI0158) and individual National Health Service Research and Development at participating centres (for list of study centres, see supplementary material).

### Procedures

In both arms, treatment was adjusted according to software based on individual predefined study algorithms. Urine samples were collected at scheduled study visits at three time points: baseline (Visit 1, study entry), Visit 3 (week 24) and final visit (Visit 6, week 48) (full details are provided in the supplementary material – Procedures). Participants attended for assessment during exacerbations with an additional urine sample collected (figure 1). Following spot urine collection, all samples were stored at –70°C within 30 min after collection at their respective clinical site. Samples underwent one freeze–thaw cycle at Covance CLS Biobank to create aliquots that were sent for eicosanoid metabolite analysis.

### Analysis of urinary eicosanoids

Urine eicosanoid metabolites were quantified using liquid chromatography coupled to tandem mass spectrometry (LC-MS/MS) [16]. A summary is provided in the supplementary material – Quantification of urinary eicosanoids*.*

### Creation of representative pathway-score variables

“Normalised pathway scores” (pathway scores) were developed by combining eicosanoid metabolites from each of the prostaglandin (PGE_2_, PGD_2_, PGF_2α_), thromboxane-A_2_, isoprostane and CysLT pathways, thereby concatenating individual metabolites from the same pathway into a single variable (supplementary figure S1). Pathway scores were calculated by z-scoring each log_2_-transformed eicosanoid metabolite concentration (to reduce skew), subtracting the mean and then dividing by the standard deviation. The mean z-score of all metabolites was then calculated within that pathway per participant.

### Statistical analysis

Descriptive statistics are presented as means, medians or counts as appropriate. Independent t-tests (normally distributed data), Mann–Whitney U-test (non-normally distributed data) and chi-squared tests (categorical data) were used to compare independent groups (“Group definitions” – supplementary material). Only one urine sample, collected at the first relevant visit, was included from each study participant in order to preserve statistical independence.

The test–retest stability of individual metabolites was estimated using the intra-class correlation (ICC) using samples from scheduled visits (sampling details are described in the supplementary materials – Statistical analysis, and figure 1)*.* These were derived using random-effect linear models with parametric bootstrapped confidence intervals (CI) [17]. To limit treatment effects, our stability analysis was limited to visits where inhaled corticosteroid (ICS) and oral corticosteroid (OCS) doses were unchanged. Samples within 14 days after an exacerbation were excluded as metabolites can be raised during exacerbations [18].

To further restrict our analysis of test–retest stability to visits with a similar clinical presentation, we conducted a sensitivity analysis restricting to visits with similar symptoms (ΔAsthma Control Questionnaire-7 (ACQ-7) score ≤0.5) and T2 biomarkers (ΔBEC ≤0.10×10^9^ cells·L^−1^, Δ*F*_ENO_ ≤10 ppb). Based upon Cicchetti criteria, the following ICC cut-offs were used: <0.40=poor, 0.40–0.59=fair, 0.60–0.74=good, 0.75–1.00=excellent [19]. Spearman's correlation was used (bootstrapped 95% CI) to calculate the association between change in eicosanoid concentrations, between baseline and Visit 3, with change in T2 biomarkers and symptoms.

When data were paired (*e.g.*, comparing changes between stable and exacerbation visits), paired t-tests (normally distributed data), Wilcoxon signed-rank tests (non-normally distributed data) and McNemar's tests (categorical data) were used to compare differences between groups. When participants had eicosanoids measured at multiple exacerbation visits, the first sample was used to preserve statistical independence. We identified visits where participants changed ICS dose or initiated/discontinued OCS to investigate the association between CS and eicosanoids. Two-tailed hypothesis tests were conducted at the 5% α-level, with 95% CIs presented throughout this analysis. Analyses were conducted under a complete-case framework using Stata version 16 (StataCorp, College Station, TX, USA).

## Results

Baseline demographics, medical history, comorbidities, lung function and treatments are summarised in table 1. Participants were predominantly female (64.8%), older (mean±sd age: 55.8 ±13.0 years), obese (mean±sd BMI: 31.6 ±7.2 kg·m^−2^) with obstructive lung function (mean±sd forced expiratory volume in 1 s (FEV_1_)/forced vital capacity (FVC): 0.65±0.12) and had multiple comorbidities including hypercholesterolaemia (17.4%) and hypertension (31.5%). Participants were predominantly T2-low (median (range) *F*_ENO_: 20 (13–29) ppb and BEC: 0.20 (0.11–0.33)×10^9^cells·L^−1^) with a significant number on maintenance OCS (37.2%) and receiving high-dose ICS treatment (mean±sd BDP equivalent: 2242±716 µg). Participants had increased symptoms (mean±sd ACQ-7: 2.0±1.2) and a poor asthma-related quality of life (mean±sd asthma quality of life questionnaire score: 4.9±1.4). Participants (n=301) provided 911 urine samples; 80 samples were excluded (figure 1). This resulted in 728 samples collected during scheduled study visits and 103 samples from exacerbation visits (figure 1).

**TABLE 1:** Baseline demographics of participants (n=298) who provided urine samples

	
**Age at inclusion years**	55.8±13.0
**Sex**	
Female	193 (64.8)
Male	105 (35.2)
**Ethnicity**	
White	276 (92.6)
Non-white	22 (7.4)
**BMI, kg·m^−2^**	31.6±7.2
**Smoking status**	
Never smoked	223 (74.8)
Ex-smoker	75 (25.2)
**Comorbidities**	
Atopic disease	205 (69.0)
History of rhinitis	205 (68.8)
History of eczema	98 (32.9)
History of nasal polyps	73 (24.5)
History of aspirin sensitivity	47 (15.8)
History of oesophageal reflux	177 (59.4)
Depression/anxiety	90 (30.2)
Hypertension	94 (31.5)
Osteoporosis/osteopenia	66 (22.1)
Hypercholesterolaemia	52 (17.4)
Diabetes	34 (11.4)
**Healthcare attendances**	
Primary care visits for asthma in the last year (any)	161 (54.0)
Rescue courses of OCS in the last year	2 (1–4)
Prior admission for asthma to a high dependency/intensive care unit	64 (21.5)
Ever been ventilated	31 (10.4)
**Lung function**	
FEV_1_ % predicted	75.5±19.3
FVC % predicted	91.1±16.9
FEV_1_/FVC	0.65±0.12
**T2 biomarkers**	
*F*_ENO_, ppb	20 (13–29)
Blood eosinophil count (×10^9^ cells·L^−1^)	0.21 (0.11–0.33)
Periostin, ng·mL^−1^	52.9±16.2
**Medications**	
Maintenance OCS	111 (37.2)
OCS dose mg	10 (5–10)
ICS dose (BDP equivalent), µg	2242±716
LAMA user	143 (48.0)
LTRA user	149 (50.0)
**Symptoms and quality of life measures**	
ACQ-7 score	2.0±1.2
AQLQ total score	4.9±1.4

Data are presented as n (%), mean±sd or median (IQR). BMI: body mass index; OCS: oral corticosteroid; FEV_1_: forced expiratory volume in 1 s; FVC: forced vital capacity; *F*_ENO_: fractional exhaled nitric oxide; ICS: inhaled corticosteroid; LAMA: long-acting muscarinic antagonists; LTRA: leukotriene-receptor antagonists; ACQ-7: asthma control quesionnaire-7; AQLQ: asthma quality of life questionnaire.

### Stability of eicosanoid levels across stable study visits

Eicosanoid pathway scores were broadly unaffected by OCS removal, ICS reduction or leukotriene-receptor antagonists (LTRA) exposure (see description in the supplementary material – Effects of asthma treatments and associated disease processes on urinary eicosanoid levels, supplementary tables S1–S3). We therefore examined the stability of eicosanoid levels across serial visits (Visit 1 (baseline), Visit 3 (week 24) and Visit 6 (week 48)) in participants where CS treatment was unchanged (n=298, samples=536) (table 2). 10 metabolites exhibited at least a “fair” degree of stability (ICC ≥0.4). The isoprostane pathway evidenced a “good” degree (ICC=0.61) of stability whereas the PGE_2_, PGD_2_, PGF_2α_, TXA_2_ and CysLT pathways evidenced a “fair” degree of stability (ICC ≥0.4) (table 2).

**TABLE 2:** Intra-class correlation (ICC) of urinary eicosanoids in participants where corticosteroid (CS) treatment was unchanged across scheduled study visits (Baseline, Visit 3 and Visit 6)

Eicosanoid	Participants	Observations	ICC (95% CI)
**PGE_2_; Pathway Normalised^#^**	298	536	0.57 (0.49–0.66)
** **TetranorPGEM	298	536	0.57 (0.49–0.66)
**PGD_2_; Pathway Normalised^#^**	298	536	0.51 (0.41–0.60)
** **2,3-dinor-11β-PGF_2α_	298	536	0.38 (0.27–0.48)
** **TetranorPGDM	298	536	0.49 (0.38–0.6)
**PGF_2α_; Pathway Normalised^#^**	298	536	0.45 (0.35–0.55)
** **PGF_2α_	298	536	0.54 (0.44–0.64)
** **TetranorPGFM	298	536	0.31 (0.20–0.42)
** **13,14-dihydro-15-ketoPGF_2α_	298	536	0.48 (0.38–0.57)
**TXA2; Pathway Normalised^#^**	298	536	0.52 (0.43–0.62)
** **11-dehydro-2,3-dinor-TXB_2_	298	536	0.39 (0.29–0.49)
** **11-dehydroTXB_2_	298	536	0.44 (0.34–0.54)
** **2,3-dinor-TXB_2_	298	536	0.48 (0.38–0.58)
**Isoprostanes; Pathway Normalised^#^**	298	536	0.61 (0.53–0.69)
** **8-iso-PGF_2α_	298	536	0.19 (0.00–0.25)
** **2,3-dinor-8-iso-PGF_2α_	298	536	0.54 (0.45–0.63)
** **5-iPF_2α_-VI	298	536	0.64 (0.57–0.72)
** **8,12-iso-iPF_2α_-VI	298	536	0.71 (0.65–0.77)
**CysLT; Pathway Normalised^#^**	298	536	0.42 (0.30–0.54)
** **LTE_4_	298	536	0.42 (0.30–0.54)

Scheduled study visits were restricted to visits where the participant was on the same CS treatment regimen. (Confidence intervals calculated for each value). The total number of participants was n=298 with 536 observations for each mediator. PGE_2_: prostaglandin-E_2_; PGD_2_: prostaglandin-D_2_; PGF**_2_**_α_: prostaglandin-F**_2α_**; TXA_2_: thromboxane; CysLT: cysteinyl-leukotriene. ^#^: Pathway Normalised – calculated mean of z-scores from analytes of the same pathway using log2-transformed concentrations of each individual analyte.

**FIGURE 2:**
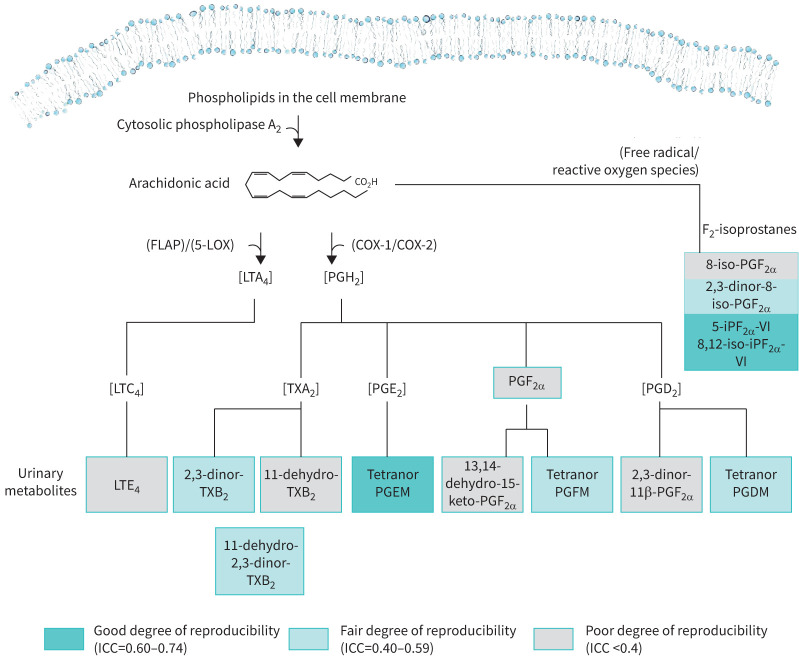
The stability of urinary eicosanoids in participants (n=63), where corticosteroid (CS) treatment was unchanged, during scheduled study visits who had stable T2 biomarkers (Δ blood eosinophil count (BEC) ≤0.10×10^9^ cells·L^−1^, Δ fractional exhaled nitric oxide (*F*_ENO_) ≤10 ppb) and stable symptoms (Δ asthma control questionnaire (ACQ-7) ≤0.5) across scheduled visits (Baseline, Visit 3 and Visit 6), based on intra-class correlation (ICC) on serial measures. Samples taken from scheduled study visits. Restricted to visits where the participants were on the same CS treatment regimen. Pairwise comparison of visits where ΔACQ-7 ≤0.5, Δ blood eosinophil count ≤0.10×10^9^ cells·L^−1^, Δ fractional exhaled nitric oxide ≤10 ppb.

We also examined stability in a participant subgroup (n=63) where CS treatment was unchanged with stable symptoms (ΔACQ-7 <0.5) and stable T2 biomarkers (ΔBEC ≤0.10×10^9^ cells·L^−1^, Δ*F*_ENO_ ≤10 ppb), between any two scheduled study visits (Visit 1, Visit 3, Visit 6). This resulted in 126 samples from 63 participants. Eight metabolites exhibited at least a “fair” degree of stability (ICC ≥0.4) in this “clinically stable” subgroup (figure 2, supplementary table S4). The PGE_2_ and isoprostane pathways demonstrated a “good” (ICC=0.64) and “fair” (ICC=0.57) degree of stability, respectively. The remainder evidenced low ICCs (ICC <0.4).

We examined the relationship between eicosanoids, T2 biomarkers and symptoms (ACQ-7) in participants on stable CS treatment from Visit 1 and Visit 3 data (supplementary table S5). The PGE_2_, PGD_2_, PGF_2α_, and TXA_2_ pathways correlated weakly with BEC (rho ≤0.22, p<0.05). The CysLT pathway correlated weakly with BEC and ACQ-7 (rho ≤0.20, p<0.05).

### Differences in eicosanoid levels in “symptom-low” and “symptom-high” participants

We compared symptom-low (ACQ-7 ≤1.5, n=115) to symptom-high (ACQ>1.5, n=183) participants, irrespective of T2 status (supplementary table S6). The isoprostane pathway score was higher in symptom-high *versus* symptom-low participants (p=0.01), with higher concentrations of 8,12-*iso*-iPF_2α_-VI and 8-*iso*-PGF_2α_ observed in symptom-high participants. When stratified for obesity, there was no difference in the pathway scores in either group; however, the concentration of 8-*iso*-PGF_2a_ was 1.75-fold higher in symptom-high participants (p=0.04) (supplementary table S7).

### Differences in eicosanoid levels in “T2-low” and “T2-high” participants

**FIGURE 3:**
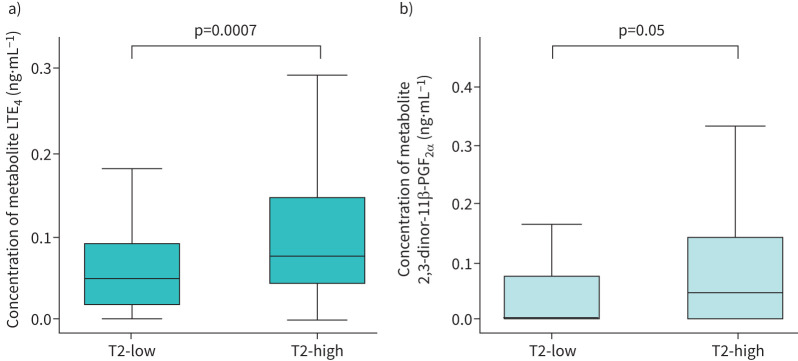
Boxplots of metabolite concentrations for “T2-high (n=161)” *versus* “T2-low (n=83)” participants, irrespective of symptom burden. Samples taken from scheduled study visits. a) Boxplot of LTE_4_ concentration in “T2-high (n=161)” *versus* “T2-low (n=83)” participants. b) Boxplot of 2,3-dinor-11β-PGF_2α_ concentration in “T2-high (n=161)” and “T2-low (n=83)” participants. T2-high: fractional exhaled nitric oxide ≥20 ppb and blood eosinophil count ≥0.15×10^9^ cells·L^−1^; T2-low: fractional exhaled nitric oxide <20 ppb and blood eosinophil count <0.15×10^9^ cells·L^−1^; LTE_4_: leukotriene-E_4_. Outlying values were excluded if they were more than 1.5 times the IQR below the first quartile (Q1) or more than 1.5 times the IQR above the third quartile (Q3).

T2-low participants (*F*_ENO_ <20 ppb and BEC <0.15×10^9^ cells·L^−1^, n=83) were compared to T2-high (*F*_ENO_ ≥20 ppb and BEC ≥0.15×10^9^ cells·L^−1^, n=161) participants, irrespective of symptom burden (supplementary table S8). LTE_4_ levels (CysLT metabolite) were higher in T2-high *versus* T2-low participants (p=0.0007) (supplementary table S8, figure 3). Although not significant, the concentration of 2,3-dinor-11β-PGF_2α_ (PGD_2_ metabolite) was raised in T2-high *versus* T2-low participants (p=0.05) (supplementary table S8, figure 3). The isoprostane pathway score was higher in T2-low *versus* T2-high obese participants; however, the difference did not reach statistical significance (p=0.05, supplementary table S9). Further sensitivity analyses were undertaken excluding participants on an LTRA (supplementary table S16) and with a diagnosis of aspirin-exacerbated respiratory disease (AERD) (supplementary table S17). Full details and description of these findings are available within the supplementary material section – Effects of asthma treatments and associated disease processes on urinary eicosanoid levels.

### Differences in eicosanoid levels in participants with symptoms dissociated with T2-biomarkers

#### “Symptom-high (ACQ-7 >1.5)/T2-low” *versus* “symptom-low (ACQ-7 **≤**1.5)/T2-low” participants

**FIGURE 4:**
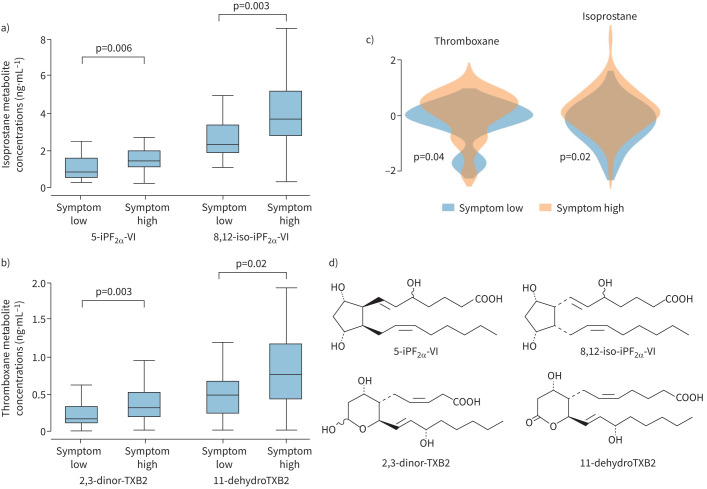
Normalised pathway scores, concentrations and chemical structures for thromboxane (TXA_2_) and isoprostane metabolites in “symptom-low (n=30)” *versus* “symptom-high (n=61)”, T2-low participants. Samples taken from scheduled study visits and restricted to participants who were T2-low. a) Boxplot for isoprostane metabolite concentrations in “symptom-low” *versus* “symptom-high”, T2-low participants. b) Boxplot for metabolite concentrations in “symptom-low (ACQ-7 ≤1.5)” *versus* “symptom-high (ACQ-7 >1.5)”, T2-low participants. c) Violin plots for thromboxane (TXA_2_) and isoprostane pathway scores in “symptom-low (ACQ-7 ≤1.5)” and “symptom-high (ACQ-7 >1.5)”, T2-low participants. d) Chemical structures of thromboxane (TXA_2_) and isoprostane metabolites in panels a and b. T2-low: fractional exhaled nitric oxide <20 ppb and blood eosinophil count 0.15×10^9^ cells·L^−1^; symptom-high: ACQ-7 >1.5; symptom-low: ACQ-7 ≤1.5. ACQ-7: asthma control quesionnaire-7. Outlying values were excluded if they were more than 1.5 times the IQR below the first quartile (Q1) or more than 1.5 times the IQR above the third quartile (Q3).

61 participants were symptom-high/T2-low (mean±sd ACQ-7: 2.6±0.8) and 30 were symptom-low/T2-low (mean±sd ACQ-7: 0.8±0.4) (table 3). The TXA_2_ (p=0.04) and isoprostane (p=0.02) pathway scores were higher in symptom-high/T2-low *versus* symptom-low/T2-low participants, with higher concentrations of 11-dehydroTXB_2_, 2,3-dinor-TXB_2_, 8,12-*iso*-iPF_2α_-VI and 5-iPF_2α_-VI observed in symptom-high/T2-low participants (p<0.05) (table 3, figure 4). Although there was no difference in pathway scores in either group following stratification for obesity, the concentration of 8,12-*iso*-iPF_2α_-VI was 1.78-fold greater in symptom-high participants (p=0.02) (supplementary table S10).

**TABLE 3:** Baseline demographics, urinary eicosanoids and T2 biomarkers for “symptom-low (ACQ-7 ≤1.5)” *versus* “symptom-high (ACQ-7 >1.5)” with T2-low status (fractional exhaled nitric oxide <20 ppb and blood eosinophil count <0.15×10^9^ cells·L^−1^)

	Symptom-low	Symptom-high	p-value
**Number of participants**	30	61	
**Sex**			0.96
Female	21 (70.0)	43 (70.5)	
Male	9 (30.0)	18 (29.5)	
**Baseline BMI, kg·m^−2^**	29.8±5.7	33.2±6.7	0.02
**Baseline FEV_1_/FVC**	0.71±0.11	0.66±0.12	0.06
**FEV_1_ % predicted**	88.5±17.0	71.7±18.6	<0.0001
**BEC (×10^9^ cells·L^−1^)**	0.11 (0.07–0.12)	0.08 (0.04–0.11)	0.013
***F*_ENO_, ppb**	14 (12–18)	13 (10–16)	0.10
**Periostin, ng·mL^−1^**	47.2±15.1	48.6±13.3	0.65
**ACQ-7 score**	0.8±0.4	2.6±0.8	<0.0001
**PGE_2_**			
PGE_2_; Pathway Normalised^#^	−0.46 (−0.93–0.35)	0.00 (−0.61–0.76)	0.10
TetranorPGEM, ng·mL^−1^	12.71 (8.09–27.97)	19.90 (10.98–41.46)	0.10
**PGD_2_**			
PGD_2_; Pathway Normalised^#^	−0.12 (−0.70–0.08)	−0.20 (−0.44–0.27)	0.30
2,3-dinor-11β-PGF_2a_, ng·mL^−1^	0.01 (0.00–0.09)	0.00 (0.00–0.06)	0.46
TetranorPGDM, ng·mL^−1^	1.93 (1.01–2.94)	2.93 (1.85–3.96)	0.014
**PGF2α**			
PGF_2α_; Pathway Normalised^#^	−0.11 (−0.57–0.37)	0.03 (−0.34–0.66)	0.18
PGF_2a_, ng·mL^−1^	1.48 (0.78–2.56)	1.87 (1.26–3.24)	0.11
TetranorPGFM, ng·mL^−1^	0.57 (0.28–2.19)	0.82 (0.16–2.94)	0.77
13,14-dihydro-15-ketoPGF_2a_, ng·mL^−1^	1.56 (1.13–2.47)	2.30 (1.74–3.23)	0.002
**TXA2**			
TXA_2_; Pathway Normalised^#^	−0.08 (−0.50–0.22)	0.28 (−0.28–0.66)	0.04
11-dehydro-2,3-dinor-TXB_2_, ng·mL^−1^	0.15 (0.08–0.25)	0.23 (0.08–0.60)	0.07
11-dehydroTXB_2_, ng·mL^−1^	0.48 (0.22–0.67)	0.76 (0.42–1.16)	0.02
2,3-dinor-TXB_2_, ng·mL^−1^	0.16 (0.10–0.33)	0.32 (0.18–0.52)	0.003
**Isoprostanes**			
Isoprostanes; Pathway Normalised^#^	−0.14 (−0.61–0.38)	0.13 (−0.27–0.66)	0.02
8-iso-PGF_2a_, ng·mL^−1^	0.08 (0.04–0.15)	0.13 (0.06–0.29)	0.09
2,3-dinor-8-iso-PGF_2a_, ng·mL^−1^	0.37 (0.16–0.57)	0.48 (0.19–1.29)	0.14
5-iPF_2a_-VI, ng·mL^−1^	0.80 (0.53–1.56)	1.40 (1.09–1.94)	0.006
8,12-iso-iPF_2a_-VI, ng·mL^−1^	2.32 (1.80–3.33)	3.66 (2.79–5.21)	0.003
**CysLT**			
CysLT; Pathway Normalised^#^	0.04 (−2.09–0.53)	0.09 (−0.43–0.45)	0.55
LTE_4_, ng·mL^−1^	0.05 (0.00–0.10)	0.05 (0.02–0.09)	0.55

Samples taken from scheduled study visits. Data are presented as counts, n (%), median (IQR) or mean±sd. Symptom-low: ACQ-7 ≤1.5; Symptom-high: ACQ-7 >1.5. ACQ-7: asthma control quesionnaire-7; BMI: body mass index; FEV_1_: forced expiratory volume in 1 s; FVC: forced vital capacity; BEC: blood eosinophil count; *F*_ENO_: fractional exhaled nitric oxide; PGE_2_: prostaglandin-E_2_; PGD_2_: prostaglandin-D_2_; PGF**_2_**_α_: prostaglandin-F**_2α_**; TXA_2_: thromboxane; CysLT: cysteinyl-leukotriene. ^#^: Pathway Normalised – calculated mean of z-scores from analytes of the same pathway using log2-transformed concentrations of each individual analyte. Restricted to participants who were T2-low (fractional exhaled nitric oxide <20 ppb and blood eosinophil count <0.15×10^9^ cells·L^−1^).

#### “Symptom-high (ACQ-7 >1.5)/T2-high” *versus* “symptom-low (ACQ-7 ≤1.5)/T2-high” participants

71 participants were symptom-low/T2-high (mean±sd ACQ-7: 0.8±0.4) and 104 were “symptom-high/T2-high” (mean±sd ACQ-7: 2.7±0.8) (supplementary table S11). There was no difference in pathway scores in either group. When stratified for obesity, there was no difference in pathway scores in either group (supplementary table S12).

### Comparison of eicosanoid levels between baseline and exacerbation visits

There were 70 reported exacerbations with eicosanoids matched to a baseline visit (supplementary table S13). Consistent with exacerbations, symptoms were increased, lung function reduced and *F*_ENO_ elevated compared to baseline. There was no difference in eicosanoid pathway scores, BEC or serum periostin during exacerbations compared to baseline.

### Comparison of eicosanoid levels between “T2-low” and “T2-high” exacerbation visits

No differences were observed in any eicosanoid levels between stable state (baseline) and exacerbation in the entire study (supplementary table S13). When stratified into T2-high (n=29) and T2-low (n=18) exacerbations, the CysLT pathway was higher in T2-high *versus* T2-low events, albeit not statistically significant (p=0.05, supplementary table S14). The CysLT pathway was more elevated during T2-high *versus* T2-low exacerbations from baseline (p=0.03), which was also observed for the PGD_2_ pathway (p=0.02), and its metabolite TetranorPGDM (p=0.02) (supplementary table S14).

During exacerbations the PGE_2_, PGD_2_, PGF_2α_ and isoprostane pathways correlated weakly (rho <0.4, p<0.05) with BEC (supplementary table S15). The TXA_2_ pathway correlated weakly with BEC and ACQ-7 (rho <0.3, p<0.05). The CysLT pathway correlated strongest with BEC (rho=0.43, p<0.05) but weakly with *F*_ENO_ and ACQ-7 (rho <0.3, p<0.05).

## Discussion

We hypothesised that elevated eicosanoids may contribute to persistent symptoms in T2-low severe asthma. Our analysis demonstrated that raised urinary isoprostane and thromboxane metabolite levels were associated with increased symptoms and poorer lung function in T2-low participants.

Study participants were predominantly female, obese, with obstructive airways disease, frequently on maintenance OCS, attended primary/secondary care often, and had several comorbidities. This patient group, predominantly T2-low but highly symptomatic, is frequently seen in severe asthma cohorts [4, 20–22]. The presence of persistent symptoms, despite suppressed T2 biology, suggests other mechanisms may be driving increased symptoms.

Eight eicosanoids, representing end-stage metabolites of the PGE_2_, PGD_2_, PGF_2_, TXA_2_ and isoprostane pathways, exhibited less between time-point variability across scheduled study visits (ICC ≥0.4) in a “clinically stable” participant subgroup (determined by symptoms, T2-biomarkers and CS treatment). In this subgroup, the isoprostane and PGE_2_ pathways exhibited a greater degree of stability (ICC ≥0.4). Previous work demonstrated that the LC-MS/MS methods employed possess analytical precision over serial measurements suggesting that sample processing, storage and measurements do not explain the between-visit variability observed [9, 16]. Consistent with previous studies, pathway scores were unaffected by CS/LTRA exposure, which suggests asthma treatment changes are unlikely to explain these differences [13, 23–25].

For the other eicosanoid pathways (PGD_2_, PGF_2α_, TXA_2_, CysLT), the temporal variability observed across serial time points (ICC <0.4) in “clinically stable” participants may reflect changes in molecular mechanisms, and processes, not directly linked to the clinical presentation of severe asthma. The changes in these metabolites may reflect residual and/or ongoing mast cell, eosinophil and platelet activity, which can be increased in numerous disease processes, including diabetes and obesity, frequently observed in patients with severe asthma [26–30]. In “clinically stable” participants, several pathways displayed increased between time-point variability, suggesting these metabolites have a complex relationship with self-reported asthma symptoms and T2 biomarkers. Subsequently, these molecular signals may follow a different kinetic onset and regulation than clinical manifestations.

A key focus of this analysis was assessing the relationship between symptoms and eicosanoid levels. As obesity is an important mediator of symptoms in severe asthma, we explored if obesity was related to eicosanoid production and mediated differences in symptoms [3]. Isoprostane levels were higher in symptom-high (ACQ-7 >1.5) *versus* symptom-low (ACQ-7 ≤1.5) participants irrespective of T2 status and associated with lower lung function in symptom-high participants. However, no difference was observed after stratifying for obesity. Isoprostanes are markers of oxidative stress and thought to increase neutrophilic inflammation by neutrophil adhesion to human venous endothelial cells and upregulating macrophage interleukin-8 expression through the activation of extracellular signal-regulated kinase 1/2 and p38 mitogen-activated protein-kinase signaling [31–33]. Neutrophilic inflammation has been suggested as a driver of symptoms in patients with refractory asthma [34]. Subsequently, isoprostanes are potentially acting as surrogate markers for neutrophilic inflammation and represent a novel pathway for monitoring symptoms [34]. However, these metabolites are systemically raised in several disease processes and may reflect metabolic dysfunction in a highly comorbid patient group [26, 27, 29, 30]. Future studies should account for comorbidities when evaluating associations with isoprostane levels.

LTE_4_ levels were higher in T2-high *versus* T2-low participants, irrespective of symptoms. Previously, raised LTE_4_ concentrations were associated with increased asthma severity, poorer lung function and T2 inflammation [13]. Although of borderline significance, LTE_4_ concentrations remained elevated in T2-high *versus* T2-low participants, having excluded those on montelukast (LTRA). In keeping with two other large severe asthma studies (UBIOPRED and SoMOSA), LTRA exposure appears not to affect metabolite levels, which suggests that remaining activation of the CysLT pathways may be occurring independently of LTRA treatment [13, 35]. Overall, our findings support previous work that increased LTE_4_ levels correlate with eosinophilic T2-related inflammation and may prove useful in identifying T2-high patients [13]. Given that AERD is associated with both eosinophilia and mast cell activation, we conducted a sensitivity analysis (excluding participants with AERD) to ensure that a diagnosis of AERD was not confounding the relationship between T2-biomarkers of inflammation and eicosanoid levels, in T2-high and T2-low participants [36]. We found that whilst the CysLT pathway score remained raised in T2-high *versus* T2-low participants, the isoprostane pathway was significantly elevated in T2-low *versus* T2- high participants. As mentioned, non-T2-related comorbidities are observed commonly in T2-low severe asthma and known to be significant drivers of oxidative stress, and this might explain increased isoprostane metabolite concentrations in this group [33].

In T2-low participants, higher TXA_2_ and isoprostane pathway scores were associated with increased symptoms. Although levels were elevated following stratification for obesity, they were no longer significant (p=0.06 and p=0.12, respectively). TXB_2_ metabolites are useful measures of *in vivo* TXA_2_ synthesis and purported to be mediators of airway inflammation in asthma [37]. These metabolites can cause bronchoconstriction through thromboxane-receptor (TP) activation [37–39]. Similarly, F_2_-isoprostanes (5-iPF_2α_-VI and 8,12-*iso*-iPF_2α_-VI) are also potent TP-activators [33, 40, 41]. TP-activation is proposed to lead to an influx of Ca^2+^ into airway smooth muscle cells, which promotes airway hyperresponsiveness and subsequent long-term airway remodeling [42–44]. Consequently, upregulation of TXA_2_ and isoprostane formation may lead to increased TP activation, which potentially explains persistent symptoms and increased disease severity in T2-low participants. However, as these pathways were unaffected by CS exposure, CS treatment is unlikely to be efficacious in reducing metabolite concentrations. Further consideration should be given to TP-receptor antagonists that may dampen symptoms associated with increased metabolite levels [45]. This should preferably be tested using a stratified approach in T2-low patients, with evidence of increased TP activity.

There were 70 exacerbation visits during the study and increased eicosanoid levels were not observed during exacerbations from baseline. Most eicosanoids correlated weakly with changes in T2 biomarkers and ACQ-7 during exacerbations, which questions the relationship between these pathways and routine measures taken at assessment following an exacerbation. Although not significant (p=0.05), LTE_4_ levels were higher during “T2-high” *versus* “T2-low” exacerbations and demonstrated a stronger correlation with BEC (rho=0.43). Interestingly, both LTE_4_ (CysLT metabolite) and the mast cell marker TetranorPGDM (PGD_2_ metabolite) were increased from baseline during T2-high *versus* T2-low exacerbations. Our findings support previous work showing that LTE_4_ was higher during emergency room presentations with acute severe asthma and decreased in the following 2 weeks, with FEV_1_ increasing from 49% to 66% in that period [46].

Our severe asthma study adopted a robust RCT design, collecting a large number of urine samples during scheduled and exacerbation study visits from well-characterised patients. Our observations were strengthened by the contemporaneous measurement of T2 biomarkers, symptoms and lung function fostering the analysis of potential relationships between eicosanoid levels and well-defined clinical characteristics. Additionally, the LC-MS/MS methodology employed provided a broad quantitative panel of eicosanoids and demonstrates analytical precision over serial measurements [9, 16]. Furthermore, we utilised multiple analyses to investigate relationships between T2 biomarkers, symptoms and eicosanoids in “clinically stable” participants. However, care should be taken when interpreting the findings of this analysis as multiple statistical tests have been utilised to standardise metabolite concentrations.

A study limitation is this analysis did not include healthy controls; however, a recent bronchial biopsy study demonstrated similar pathology in participants with T2-high and T2-low severe asthma, including elevated sputum LTE_4_ and PGD_2_, in both groups, *versus* healthy controls [47]. This suggests residual disease expression in T2-low severe asthma (persistent symptoms, impaired lung function, non-T2 exacerbations) may be driven by ongoing mast cell activation and eicosanoid production, which is supported by other studies demonstrating the presence of mast cell activation in severe asthma [48, 49]. Notably, asthma is a heterogeneous disease process characterised by a phenotypically diverse patient population who often experience significant comorbidities and are exposed to high levels of treatment [2]. Multiple disease processes as well as specific asthma medications may be potentially influencing eicosanoid metabolite concentrations. Given the degree of comorbidity observed in this cohort, it is difficult to control for multiple residual confounding factors. Future studies may consider collecting urine samples at regular time points to assess changes in metabolite concentrations in different groups of patients with asthma. Another limitation of this study is that *F*_ENO_ was not particularly elevated in T2-high participants (median *F*_ENO_: 28 ppb), irrespective of symptom burden. This was due to the original study design enriching for T2-low participants with a *F*_ENO_ <45 ppb [14]. Future studies may consider recruiting participants with a *F*_ENO_ in excess of 45 ppb to further investigate the relationship between metabolite concentrations and T2 biology. Nevertheless, a *F*_ENO_ of ≥25 ppb and a BEC >0.15×10^9^ cells·L^−1^ is associated with increased annual exacerbation rates in patients with two or more clinical risk factors when compared to composite T2-low patients without clinical risk factors [50].

A limitation of the exacerbation analysis is that eicosanoids are rapidly produced and released upon cellular activation and are best collected within 6 h of symptom onset during exacerbations [51]. Given the study design, participants may have attended beyond this window, which may have exceeded the timespan of eosinophil, mast cells and basophil granulation.

### Conclusion

Higher urinary isoprostane and TXA_2_ metabolite levels were associated with increased symptoms in T2-low participants, and our findings suggest that these eicosanoids can discriminate symptoms in this understudied asthma subgroup. These metabolites are potentially promoting airway hyperresponsiveness and long-term airway remodeling, *via* the thromboxane receptor, and might explain persistent symptoms in T2-low patients. Further research is needed to understand the role of eicosanoids in T2-low severe asthma and specifically interventions to perturb these metabolic pathways within a clinical trial setting.

## Supplementary material

**Please note:** supplementary material is not edited by the Editorial Office, and is uploaded as it has been supplied by the author. Supplementary material 01089-2024.SUPPLEMENT
