# Supplementary material for: Urinary thromboxane and isoprostane levels are elevated in symptom-high T2-biomarker-low severe asthma
Source: ERJ Open Res. 2025 Aug 26;11(4):01089-2024. doi: 10.1183/23120541.01089-2024 (PMC12378740; doi:10.1183/23120541.01089-2024)
Supplement: Supplementary file 1 [file 01089-2024.SUPPLEMENT.pdf]

Supplementary material

Urinary thromboxane and isoprostane levels are elevated in symptom-high T2-biomarker low severe asthma

Matthew Chad Eastwood, John Busby, Johan Kolmert, Javier Zurita, Sven-Erik Dahlén, Pamela Jane McDowell, Judy Bradley, David J Jackson, Ian D Pavord, Ratko Djukanovic, Joesph R Arron, Peter Bradding , Chris E Brightling, Rekha Chaudhuri, Douglas Cowan, Stephen J Fowler, Timonthy C Hardman, Celcile T J Holweg, James Lordan, Adel H Mansur, Dougals S Robinson, Craig E Wheelock, Liam G Heaney, on behalf of the investigators for the UK MRC Refractory Asthma Stratification Program (RASP-UK)

Index of tables and figures

Tables		Pages
**Table S1**	Demographic, T2-biomarkers and urinary eicosanoid concentrations in participants “Off” and “On” oral corticosteroid (OCS)	15-17
**Table S2**	Demographic, T2-biomarkers and urinary eicosanoid concentrations in participants on lower and higher dose Inhaled corticosteroid (ICS)	18-20
**Table S3**	Demographic, T2-biomarkers and urinary eicosanoid concentrations in participants receiving a Leukotriene receptor antagonist versus those not receiving a Leukotriene receptor antagonist (LTRA)	21
**Table S4**	Sensitivity analysis showing Intra-cross correlation (ICC) between urinary eicosanoids in participants where corticosteroid (CS) treatment was unchanged and who had stable symptoms and T2-biomarkers across scheduled study visits.	22
**Table S5**	Spearman rank correlation (r) between urinary eicosanoids and asthma control questionnaire-7 (ACQ-7), fractional exhaled nitric-oxide (FeNO) and blood eosinophil count (BEC) in all participants who were on stable CS treatment at scheduled study visits 1 and 3.	23
**Table S6**	Demographics, biomarkers and urinary eicosanoid concentrations in “symptom-high” versus “symptom-low” participants in the entire study cohort	24
**Table S7**	Demographics, biomarkers and urinary eicosanoid concentrations in “symptom-high” versus “symptom-low” participants with BMI ≥30kg/m^2 (obese)	25
**Table S8**	Demographics, T2-biomarkers and urinary eicosanoid concentrations in“T2-low” versus “T2-high” participants in the entire study	26

**Table S9**	Demographics, T2-biomarkers and urinary eicosanoid concentrations in “T2-high versus “T2-low” participants with BMI ≥30kg/m^2 (obese)	27
**Table S10**	Demographics, T2-biomarkers and urinary eicosanoid concentrations in “symptom-high” versus “symptom-low” participants with T2-low status with BMI ≥30kg/m^2 (obese)	28
**Table S11**	Demographics, T2-biomarkers and urinary eicosanoid concentrations in “symptom-low” versus “symptom-high” participants with T2-high status	29
**Table S12**	Demographics, T2-biomarkers and urinary eicosanoid concentrations in “symptom-low” versus “symptom-high” participants with T2-high status and BMI ≥30kg/m^2 (obese)	30
**Table S13**	Demographics, T2-biomarkers and urinary eicosanoid concentrations at baseline and during an exacerbation in the overall study cohort	31-33
**Table S14**	Subgroup analysis of demographics, T2-biomarkers and urinary eicosanoid concentrations of participants who were “T2-high” and “T2-low” during an exacerbation	34-36
**Table S15**	Spearman rank correlation (r) between urinary eicosanoid concentrations, blood eosinophil count (BEC), fractional exhaled nitric-oxide (FeNO) and Asthma control questionnaire-7 (ACQ-7) during an exacerbation	37
**Table S16**	Sensitivity analysis showing demographics, T2-biomarkers and urinary eicosanoid concentrations in T2-low and T2-high participants having excluded participants on an leukotriene receptor antagonist (LTRA)	38
**Table S17**	Sensitivity analysis showing demographics, T2-biomarkers and urinary eicosanoid concentrations in T2-low and T2-high participants having excluded participants with aspirin exacerbated respiratory disease (AERD)	39
**Figures**		
**Figure S1**	Flow diagram showing metabolites used to calculate “normalised pathway scores”	12

List of participating clinical centres

NHS Clinical Centres with a dedicated tertiary care difficult asthma service which recruited to the study

- Belfast Health AND Social Care Trust
- Oxford University Hospitals NHS Trust
- Glenfield Hospital, University Hospitals of Leicester NHS Trust
- Wythenshawe Hospital, University Hospitals of South Manchester NHS Trust
- University Hospital Southampton NHS Foundation Trust
- Royal Brompton AND Harefield NHS Foundation Hospital
- King's College Hospital NHS Foundation Trust
- Nottingham University Hospitals NHS Foundation Trust
- Sheffield Teaching Hospitals NHS Foundation Trust
- Gartnavel and Stobhill/Glasgow Royal Infirmary Hospitals, Greater Glasgow Health Board
- Heartlands Hospital, Heart of England NHS Foundation Trust
- Freeman Hospital, Newcastle upon Tyne NHS Foundation Trust

List of industrial partners

- Unit of Integrative Metabolomics, Institute of Environmental Medicine, Karolinska Institute, Stockholm, Sweden.
- GSK
- Hoffman la Roche / Genentech Inc
- Amgen

- Astra Zeneca / Medimmune
- Boehringer Ingelheim
- Jannsen
- Circassia
- Vitalograph

Abbreviations: LC-MS/MS (liquid chromatography with tandem mass spectrometry), ACQ-7 (asthma control questionnaire-7) , CS (corticosteroid), OCS (oral corticosteroid) , ICS (inhaled corticosteroid), FeNO (fractional exhaled nitric oxide), parts per billion (ppb), BEC (blood eosinophil count), NHS (national health service research), T2-Biomarker High (T2-high), FEV_1 (forced expiratory volume in 1 second), FVC (forced vital capacity) LABA (long acting beta agonist), FP (fluticasone propionate), AQLQ (asthma quality of life questionnaire), BMI (body mass index*),* TX (thromboxane), TXA_2 (thromboxane-A2), TXB_2 (thromboxane-B2), CysLT (cysteinyl-leukotriene), PGI_2 (prostaglandin-I_2), LTE_4 (leukotriene-E_4), TP (thromboxane receptor), PGD_2 (prostaglandin-D_2), PGE_2 (prostaglandin-E2), $PGF_2\alpha$ (prostaglandin-F2α), eCRF (electronic case report form), RCT (randomized control trial), ICC (intra-class correlation coefficients), SPE (polymeric solid-phase extraction) CV (coefficient of Variation), LQC (low quality control), HQC (High quality control), S/N (signal-to-noise), LTRA (leukotriene receptor antagonist), AERD (aspirin exacerbated respiratory disease)

Study subject inclusion and exclusion criteria

The full study protocol has been previously published [E1]

Inclusion criteria

Participants must meet the following criteria at screening for study entry (participants can be rescreened for study entry up to 3 times):

1. Age ≥ 18 and ≤ 80 years at screening visit
2. Able and willing to provide written informed consent and to comply with the study protocol
3. Baseline fractional exhaled nitric oxide (FeNO)< 45 ppb at screening *
4. Severe asthma confirmed after assessment by an asthma specialist. Diagnosed with asthma at least 12 months prior to screening
5. Current asthma treatment with long-acting beta agonist (LABA) plus high doses of inhaled corticosteroids (ICS) (≥1000 µg fluticasone propionate [FP]daily or equivalent)
6. Participants on an ICS/LABA single inhaler strategy must be switched to fixed dosing ICS/LABA for 4 weeks prior to screening
7. Documented history of reversibility of ≥12% change in FEV_1 within the past 24 months or during screening period, as demonstrated by:
    - Documented airflow obstruction (forced expiratory volume in 1 second/forced vital capacity (FEV_1/FVC) <70%), where FEV_1 has varied by ≥12% either spontaneously or in response to oral corticosteroid (OCS) therapy or bronchodilators either between or during clinic visits

    Or

- A 20% drop in FEV_1 (PC_{20}) to methacholine <8 mg/mL or a 15% fall in FEV_1 (PD_{15}) after inhaling a cumulative dose of mannitol of ≤635 mg indicating the presence of airway hyperresponsiveness. If sites customarily use histamine to perform tests of airway responsiveness, this may be used in place of methacholine. **

Exclusion criteria

Participants who meet any of the following criteria will be excluded from study entry

1. Acute exacerbation requiring oral corticosteroids in previous 4 weeks before screening (subjects were eligible for rescreening and inclusion).
2. If recently commenced on a leukotriene receptor antagonist (LTRA) or theophylline, stable on treatment for 4 weeks prior to screening
3. Current self-reported history of smoking (including electronic inhaled nicotine products) or former smoker with a smoking history of >15 pack-years
    a. A current smoker is defined as someone who has smoked one or more cigarettes per day (or marijuana or pipe or cigar) for ≥ 30 days within the 24 months prior to the screening visit (Day –14) and / or cotinine positive at screening
    b. Any individual who smokes (cigarettes, marijuana, pipe, or cigar) occasionally, even if for < 30 days within the 24 months prior to the screening visit (Day –14), must agree to abstain from all smoking from the time of consent through completion of study

   c. A former smoker is defined as someone who has smoked one or more cigarettes per day (or marijuana or pipe or cigar) for ≥ 30 days in his or her lifetime (as long as the 30-day total did not include the 24 months prior to the screening visit [Day –14]).
   d. A pack-year is defined as the average number of packs per day times the number of years of smoking.

4. Known current malignancy or current evaluation for a potential malignancy or history of malignancy within 5 years prior to baseline, with the exception of basal-cell and squamous-cell carcinomas of the skin and carcinoma *in situ* of the cervix uteri that have been excised and cured.
5. Known severe or clinically significant immunodeficiency, including, but not limited to, human immunodeficiency virus (HIV) infection or currently receiving or have historically received intravenous immunoglobulin for treatment for immunodeficiency.
6. Other clinically significant medical disease or uncontrolled concomitant disease despite treatment that is likely, in the opinion of the investigator, to require a change in therapy or impact the ability to participate in the study
7. History of current alcohol, drug, or chemical abuse or past abuse that would impair or risk the subject’s full participation in the study, in the opinion of the investigator
8. Current use of an immunomodulatory/immunosuppressive therapy or past use within 3 months or five drug half-lives (whichever is longer) prior to the screening visit

9. Use of a biologic therapy including Omalizumab at any time during the 6 months prior to the screening visit.

10. Bronchial-thermoplasty within prior 6 months of the screening visit

11. Initiation of or change in allergen immunotherapy within 3 months prior to the screening visit.

12. Treatment with an investigational agent within 30 days of the screening visit (or five half-lives of the investigational agent, whichever is longer).

13. Female participants who are pregnant or lactating.

*Fractional Exhaled Nitric-Oxide (FeNO) was measured as per the Official American Thoracic Society (ATS) Clinical Practice Guideline Interpretation of FeNO for Clinical Applications 2011 [E2].

** Spirometry was conducted according to the ATS/European Respiratory Society guidelines with Global Lung Function 2012 equations used to calculate FEV_1 and FVC predictive values [E3, E4].

Procedures:

Within this randomized control trial (RCT), participants in both the biomarker-directed care arm and the symptom-based arm received instructions with treatment being adjusted according to software based on individual predefined study algorithms, as previously published [E1]. Asthma control questionnaire-7 (ACQ-7) scores, post-bronchodilator FEV_1, FeNO, periostin (entered automatically by a central laboratory within 3–5 days of sample

collection) and blood eosinophil count [BEC] (within 24 hours of collection) were recorded in the electronic case report form (eCRF) [E1]. Participants were blinded to both strategies, and this blinding was shown to be effective at the end of the trial [E5]. Participants were given recommendations to increase, decrease or maintain, in both arms, based on trial algorithms [E1, E5]. Participants continued to follow their self-management plan during an asthma exacerbation [E1, E5]. During an exacerbation, adjustment to background treatment and any planned therapy adjustments was delayed until the next scheduled visit [E1]. Participants were assessed by the clinical investigator if there was any concern about continued poor asthma control, exacerbations, or treatment adjustment [E1, E5]. Final assessments were conducted at week 48 (visit 6) [E1, E5].

Quantification of urinary eicosanoids

Laboratory procedure for urinary eicosanoid analysis:

A total of 911 samples were analysed in 11 batches. Frozen urine (-80 ℃) was thawed over-night at 4 ℃, vortex and centrifuged 5 min at 1500 g. Prior to analysis, samples were randomized within each subject as per sample visit. All samples from a given individual subject were analysed in the same batch-plate. From each sample, 300 µL urine was transferred to a deep 96-well plate and 10 µL IS-mix (concentration 29-1459 ng/L, varying depending upon the individual response factor of the deuterated analytes in the electrospray process) and 1500 µL dilution solvent (0.12% acetic acid in water) was added. A low- and high-quality control (QC) urine sample pool was created from five healthy humans. Eicosanoid metabolite standards were spiked into the pool in the concentration range; 0.4-220 ng/mL for the low QC (LQC) pool and 3.6-719 ng/mL for the high QC (HQC) pool. The endogenous response signal of each analyte in the native QC pool samples was used to

guide the amounts spiked in so that the LQC signal was close to the lower quartile range of healthy human urine and the HQC signal was close to the concentration near the upper quartile range. In each extracted SPE plate, 3 LQC and 3 HQC samples were included and processed together with the study samples.

Eicosanoid metabolites were extracted using a hydrophobic interaction on a polymeric solid-phase extraction (SPE) plate (Evolute ABN, 30 mg). Reconstitution of dried extracts was performed using 100 µL of MeOH:H2O (1:1) followed by a 0.2 µm filter clean-up step. A volume of 7.5 µL was then injected onto the liquid chromatography with tandem mass spectrometry (LC-MS/MS) system (Acquity UPLC coupled to a Xevo TQ-XS, Waters, Milford, US). Throughout each run sequence, two batches of 90 samples each, LQC and HQC samples were injected at regular intervals from the beginning, until the end of each run. Calibration curves were injected in the beginning and at the end of each run. Prior to starting each batch, a "System Suitability Test" was run to check instrument performance.

To quantify the complete panel of included eicosanoid metabolites, each batch of samples was analysed twice, firstly using a 21 min LC-MS/MS method to quantify 16 eicosanoid metabolites. Secondly, the extracted urine samples were derivatised in vial using methoxyamine to stabilise cyclic and/or tautomeric analytes by attaching an amine-linked methyl group (N-OCH_3-group) on the C15-carbonyl position. The analytes quantified by methoxyamine derivatisation were; 2,3-dinor-TXB_2, TXB_2 and 2,3-dinor-6-keto-$PGF_{1\alpha}$. The total run time for the derivatised method was 7 min. Due to the exploratory nature of this study concentrations of urinary eicosanoids reported had an absolute peak area with

minimum tolerance of signal-to-noise (S/N) ratio ≥3. All analyte peaks were manually inspected. Thereafter, concentrations (ng/mL or pg/mL) were dilution-adjusted and normalized to their measured specific gravity. Following specific gravity measurement (Digital Urine Specific Gravity Refractometer UG-α; Atago Co. ltd. Tokyo, Japan) the derived refractive index was used to calculate the SG dilution factor according to the following equation:

Concentration corrected = Concentration measured * (1.020 – 1)/(Specific Gravity – 1)

Data pre-processing:

Following the established S/N criterion of keeping data with S/N≥3, three eicosanoid metabolites demonstrated lower degree of overall detection among the 911 study samples. As such, several metabolites were excluded from this analysis due to a number of reasons. The PGI_2-pathway metabolite (Prostaglandin-I_2), 2,3-dinor-6-keto-$PGF_{1\alpha}$, was excluded due to a low degree of detection. Parent metabolites Prostaglandin-E_2 (PGE_2) and Thromboxnae-B_2 (TXB_2) were also excluded, as these compounds when present in urine, primarily originate from the kidneys rather than representing pathophysiological processes that occur in lung tissue (E6, E7). Initially, TetranorPGJM and TetranorPGAM were included to increase the metabolic coverage but these were excluded as they have no isotopically labelled internal standard available which led to a higher coefficient of variation (CV).

Figure S1: Flow diagram showing metabolites used to calculate "normalised pathway scores"

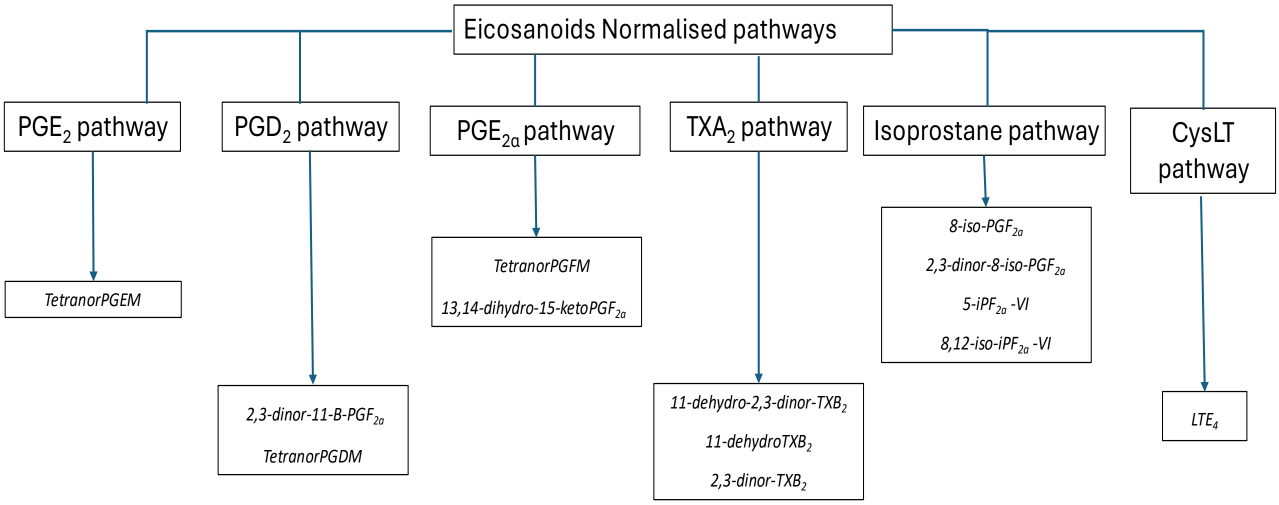

Group definitions:

T2-Biomarker low (T2-low) participants were defined as participants with a BEC<0.15 x10^9 cells/L and FeNO<20 ppb, while T2-biomarker high (T2-high) participants were defined as BEC≥0.15 x10^9 cells/L and FeNO≥20 ppb. Symptom-high was defined as an ACQ-7>1.5 and symptom-low as an ACQ-7≤1.5.

Statistical analysis:

Paired and individual samples were used in the calculation of the intra-class correlation coefficient (ICC). 62 participants had samples available from visit-1, visit-3 and visit-6. 49

participants had samples available from visits 1 and 3. 27 participants had samples available from visits 1 and 6. 38 participants had samples available from visits 3 and 6. In total, 176 participants contributed paired samples used in the calculation on the ICC for participants on stable CS treatment. 122 participants contributed a single sample. 75 samples were available from visit 1, 16 samples available from visit 3 and 31 samples available from visit 6. In total, 298 participants contributed samples (n=536) used in the calculation of the ICC in participants on stable CS treatment from scheduled study visits. Further details can be found in figure 1.

Effects of asthma treatments and associated disease processes on urinary eicosanoid levels

a) *Change in eicosanoids in participants who changed corticosteroid dose*

46 participants were weaned off maintenance OCS by the end of the study. Pathway scores were compared 'On' and 'Off' OCS (Table S1). The median [range] OCS dose was 5 (5–8) mg 'On' OCS. BEC was higher 'Off' than 'On' OCS, consistent with OCS withdrawal (0.32 vs 0.20 $x10^9$ cells/L, P<0.0001). Participants on low-dose ICS had a higher BEC, FeNO and periostin relative to those on high-dose ICS [n=55] (Table S2). Pathway scores were unaffected by removal of OCS or ICS reduction.

b) *Differences in eicosanoids in participants receiving a Leukotriene-receptor antagonists (LTRA) versus not receiving an LTRA*

142 participants receiving an LTRA were compared to 147 not receiving an LTRA. There was no difference in pathway scores between either group (Table S3, supplementary material).

c) *Differences in eicosanoids in T2-low and T2- high participants, regardless of symptom burden, having excluded participants receiving a Leukotriene-receptor antagonist (LTRA)*

87 participants were T2-high and 36 were T2-low having excluded participants receiving an LTRA at baseline (Table S16). Although statistical significance was borderline, the CysLT pathway score remained raised in T2-high compared to T2-low participants (P=0.05) having excluded participants receiving an LTRA.

d) *Differences in eicosanoids in T2-low and T2-high participants, regardless of symptom burden, having excluded participants with aspirin exacerbated respiratory disease (AERD)*

138 participants were T2-high and 67 were T2-low after excluding participants who had a diagnosis of AERD (Table S17). Notably, the CysLT pathway score remained significantly raised in T2-high compared to T2-low participants (P=0.003). The isoprostane pathway was raised in T2-low versus T2-high participants (P=0.03) after excluding participants with AERD.

Table S1: Demographics, T2-biomarkers and urinary eicosanoid concentrations in participants (n=46) "On" and "Off" oral corticosteroid (OCS)

	**Off OCS**	**On OCS**	**P-value**
**Gender**			1.00
Female	29 (63.0%)	29 (63.0%)	
Male	17 (37.0%)	17 (37.0%)	
**baseline BMI (kg/m2)**	31.4 (8.0)	31.4 (8.0)	
**% Predicted FEV$_1$**	73.9 (18.6)	75.0 (18.4)	0.69
**BEC (x10^9 cells/L)**	0.32 (0.25,0.54)	0.20 (0.11,0.34)	<0.0001
**Absolute difference from baseline in BEC (x10^9 cells/L)**		-0.10 (-0.23,-0.01)	
**FeNO (ppb)**	28 (21,36)	26 (15,33)	0.07
**Absolute difference from baseline in FeNO (ppb)**		-3 (-12,5)	
**Periostin (ng/mL)**	59.0 (16.0)	54.8 (14.3)	0.01
**Absolute difference from baseline in Periostin (ng/mL)**		-3.9 (-9.6,-0.3)	
**ACQ-7 Score**	2.1 (1.2)	1.9 (1.2)	0.24
**Absolute difference from baseline in ACQ-7 Score**		-0.2 (-0.7,0.3)	
**OCS**	0 (0.0%)	46 (100.0%)	<0.0001
**OCS Dose (mg)**	0 (0,0)	5 (5,8)	<0.0001
**ICS Dose (µg)**	2000 (2000,2000)	2000 (2000,2000)	0.57
**PGE_2**			
**PGE_2; Pathway Normalised***	0.16 (-0.66,0.95)	0.15 (-0.89,0.86)	0.19
Difference from baseline in PGE_2; Pathway Normalised		-0.20 (-0.62,0.24)	
TetranorPGEM (ng/mL)	23.21 (10.51,49.89)	22.98 (8.40,45.79)	0.62
Absolute difference from baseline in TetranorPGEM (ng/mL) †		-2.13 (-12.42,11.19)	
**PGD_2**			
**PGD_2; Pathway Normalised***	-0.20 (-0.74,0.56)	-0.11 (-0.78,0.33)	0.74
Difference from baseline in PGD_2; Pathway Normalised		-0.05 (-0.61,0.57)	
2,3-dinor-11β-$PGF_{2α}$ (ng/mL)	0.00 (0.00,0.14)	0.00 (0.00,0.09)	0.82
Absolute difference from baseline in 2,3-dinor-11β-$PGF_{2α}$ (ng/mL) †		0.00 (-0.05,0.06)	

TetranorPGDM (ng/mL)	2.77 (1.49,3.94)	2.70 (1.42,4.66)	0.50
Difference from baseline in tetranorPGDM (ng/mL) †		-0.23 (-1.18,0.92)	
**$PGF_{2\alpha}$**			
**$PGF_{2\alpha}$; Pathway Normalised***	0.01 (-0.61,0.52)	-0.01 (-0.48,0.53)	0.55
Difference from baseline in $PGF2_{\alpha}$; Pathway Normalised		0.04 (-0.21,0.35)	
PGF_{2a} (ng/mL)	1.78 (1.10,3.34)	2.14 (1.14,2.95)	0.79
Absolute difference from baseline in PGF_{2a} (ng/mL) †		0.17 (-0.81,0.86)	
TetranorPGFM (ng/mL)	0.90 (0.21,2.49)	0.91 (0.19,1.93)	0.65
Absolute difference from baseline in TetranorPGFM (ng/mL) †		-0.02 (-0.59,0.30)	
13,14-dihydro-15-ketoPGF_{2a} (ng/mL)	1.65 (1.06,2.97)	2.10 (1.27,2.76)	0.87
Absolute difference from baseline in 13,14-dihydro-15-ketoPGF_{2a} (ng/mL) †		0.10 (-0.74,0.56)	
**TXA_2**			
**TXA_2; Pathway Normalised***	0.06 (-0.69,0.45)	0.04 (-0.82,0.48)	0.91
Difference from baseline in TXA_2; Pathway Normalised		-0.06 (-0.29,0.39)	
11-dehydro-2,3-dinor-TXB_2 (ng/mL)	0.15 (0.05,0.39)	0.15 (0.01,0.42)	0.87
Absolute difference from baseline in 11-dehydro-2,3-dinor-TXB_2 (ng/mL) †		0.00 (-0.17,0.19)	
11-dehydroTXB_2 (ng/mL)	0.67 (0.38,0.95)	0.63 (0.36,0.97)	0.93
Absolute difference from baseline in 11-dehydroTXB_2 (ng/mL) †		-0.06 (-0.30,0.35)	
2,3-dinor-TXB_2 (ng/mL)	0.27 (0.11,0.46)	0.26 (0.15,0.41)	0.99
Absolute difference from baseline in 2,3-dinor-TXB_2 (ng/mL) †		0.00 (-0.18,0.17)	
**Isoprostanes**			
**Isoprostanes; Pathway Normalised***	-0.06 (-0.50,0.48)	0.12 (-0.44,0.40)	0.12
Difference from baseline in Isoprostanes; Pathway Normalised†		0.21 (-0.18,0.47)	
8-iso-PGF_{2a} (ng/mL)	0.10 (0.05,0.28)	0.12 (0.07,0.20)	0.63
Absolute difference from baseline in 8-*iso*-PGF_{2a} (ng/mL) †		0.00 (-0.14,0.09)	
2,3-dinor-8-iso-PGF_{2a} (ng/mL)	0.30 (0.14,0.77)	0.31 (0.12,0.64)	0.69
Absolute difference from baseline in 2,3-dinor-8-*iso*-PGF_{2a} (ng/mL) †		0.00 (-0.17,0.16)	
5-iPF_{2a}-VI (ng/mL)	1.05 (0.68,1.61)	1.23 (0.72,1.66)	0.05
Absolute difference from baseline in 5-iPF_{2a}-VI (ng/mL) †		0.13 (-0.09,0.41)	
8,12-iso-iPF_{2a} -VI (ng/mL)	2.78 (1.90,4.10)	3.11 (1.89,4.21)	0.42

Absolute difference from baseline in in 8,12-*iso*-iPF$_{2\alpha}$-VI (ng/mL) †		0.31 (-1.02,0.69)	
**CysLT**			
**CysLT; Pathway Normalised***	0.31 (0.08,0.73)	-0.00 (-0.38,0.60)	0.12
Difference from baseline in CysLT; Pathway Normalised		-0.01 (-0.48,0.18)	
LTE$_4$ (ng/mL)	0.07 (0.05,0.14)	0.04 (0.02,0.12)	0.36
Absolute difference from baseline in LTE$_4$ (ng/mL) †		-0.00 (-0.05,0.03)	

*Samples taken from scheduled study visits. Values presented as percentages (%) Means (SD) Median (IQR). *Pathway Normalised: calculated mean of z-scores from analytes of the same pathway using log2-transformed concentrations of each individual analyte. †Absolute differences from baseline: The values reported are the differences in the urinary eicosanoid concentrations "on" and "off" OCS treatment. Abbreviations: blood eosinophil count (BEC), fractional exhaled nitric-oxide (FeNO), forced expiratory volume in 1 second (FEV$_1$), forced vital capacity (FVC), asthma control quesionnaire-7 (ACQ-7), body mass index (BMI), prostaglandin-E$_2$ (PGE$_2$), prostaglandin-D$_2$ (PGD$_2$), prostaglandin-F$_{2\alpha}$ (PGF$_{2\alpha}$), thromboxane (TXA$_2$), cysteinyl-leukotriene (CysLT)*

Table S2: Demographic, T2-biomarkers and urinary eicosanoid concentrations in participants (n=55) on lower and higher dose Inhaled Corticosteroid (ICS)

	Lower ICS	Higher ICS	P-value
**Gender**			1.00
Female	34 (61.8%)	34 (61.8%)	
Male	21 (38.2%)	21 (38.2%)	
**Depression / Anxiety**	13 (23.6%)	13 (23.6%)	1.00
**baseline BMI (kg/m2)**	31.1 (6.4)	31.1 (6.4)	
**% Predicted FEV_1**	77.0 (20.2)	78.8 (19.5)	0.15
**BEC (x10^9 cells/L)**	0.17 (0.10,0.32)	0.14 (0.10,0.25)	0.007
Absolute difference from baseline in BEC (x10^9 cells/L)		-0.02 (-0.06,0.02)	
**FeNO (ppb)**	18 (14,26)	15 (12,22)	0.02
Absolute difference from baseline in FeNO (ppb)		-1 (-9,2)	
**Periostin (ng/mL)**	47.3 (18.5)	44.7 (15.8)	0.02
Absolute difference from baseline in Periostin (ng/mL)		-1.9 (-4.3,1.8)	
**ACQ-7 Score**	1.7 (1.1)	1.5 (1.1)	0.15
Absolute difference from baseline in ACQ-7 Score		-0.1 (-0.6,0.1)	
**OCS**	0 (0.0%)	0 (0.0%)	
**OCS Dose (mg)**	0 (0,0)	0 (0,0)	
**ICS Dose (µg)**	1000 (500,1000)	2000 (2000,2400)	<0.0001
**PGE_2**			
**PGE_2; Pathway Normalised***	-0.04 (-0.69,0.46)	-0.46 (-0.94,0.31)	0.14
Difference from baseline in PGE_2; Pathway Normalised		-0.20 (-0.61,0.35)	
TetranorPGEM (ng/mL) †	19.08 (10.16,30.99)	12.73 (7.96,26.91)	0.32
Absolute difference from baseline in TetranorPGEM (ng/mL) †		-1.85 (-9.45,6.85)	
**PGD_2**			
**PGD_2; Pathway Normalised***	-0.02 (-0.61,0.48)	-0.04 (-0.69,0.51)	0.84
Difference from baseline in PGD_2; Pathway Normalised		0.03 (-0.41,0.41)	
2,3-dinor-11β-$PGF_2α$ (ng/mL)	0.02 (0.00,0.11)	0.04 (0.00,0.12)	0.39
Absolute Diff from baseline in 2,3-dinor-11β-PGF_{2a} (ng/mL) †		0.00 (-0.02,0.06)	

TetranorPGDM (ng/mL)	2.40 (1.73,3.62)	2.32 (1.49,3.53)	0.95
Absolute difference from baseline in TetranorPGDM (ng/mL) †		0.09 (-0.82,0.85)	
**$PGF_{2\alpha}$**			
**$PGF_{2\alpha}$; Pathway Normalised***	-0.03 (-0.40,0.24)	-0.05 (-0.51,0.30)	0.42
Difference from baseline in $PGF_{2\alpha}$; Pathway Normalised		-0.05 (-0.39,0.20)	
PGF2a (ng/mL)	1.92 (1.23,2.78)	1.45 (0.84,2.46)	0.02
Absolute difference from baseline in PGF_{2a} (ng/mL) †		-0.29 (-1.13,0.29)	
TetranorPGFM (ng/mL)	0.52 (0.25,1.16)	0.56 (0.33,1.57)	1.00
Absolute difference from baseline in TetranorPGFM (ng/mL) †		0.00 (-0.58,0.61)	
13,14-dihydro-15-ketoPGF_{2a} (ng/mL)	1.80 (1.29,2.65)	1.66 (1.25,2.64)	0.39
Absolute difference from baseline in 13,14-dihydro-15-ketoPGF_{2a} (ng/mL) †		-0.00 (-0.66,0.46)	
**TXA_2**			
**TXA_2; Pathway Normalised***	0.02 (-0.55,0.39)	0.06 (-0.50,0.45)	0.46
Difference from baseline in TXA_2; Pathway Normalised		0.03 (-0.24,0.55)	
11-dehydro-2,3-dinor-TXB_2 (ng/mL)	0.17 (0.07,0.37)	0.20 (0.10,0.44)	0.67
Absolute difference from baseline in 11-dehydro-2,3-dinor-TXB_2 (ng/mL) †		0.03 (-0.11,0.17)	
11-dehydroTXB_2 (ng/mL)	0.59 (0.39,0.93)	0.57 (0.38,0.92)	1.00
Absolute difference from baseline in 11-dehydroTXB_2 (ng/mL)		-0.01 (-0.22,0.20)	
2,3-dinor-TXB_2 (ng/mL) †	0.24 (0.11,0.42)	0.23 (0.12,0.42)	0.55
Absolute difference from baseline in 2,3-dinor-TXB_2 (ng/mL) †		0.00 (-0.07,0.16)	
**Isoprostanes**			
**Isoprostanes; Pathway Normalised***	-0.18 (-0.68,0.28)	0.01 (-0.45,0.47)	0.56
Difference from baseline in Isoprostanes; Pathway Normalised		0.05 (-0.28,0.38)	
8-isoPGF_{2a} (ng/mL)	0.08 (0.00,0.15)	0.11 (0.06,0.17)	0.35
Absolute difference from baseline in 8-*iso*-PGF_{2a} (ng/mL)		0.00 (-0.03,0.08)	
2,3-dinor-8-iso-PGF_{2a} (ng/mL) †	0.31 (0.10,0.49)	0.36 (0.15,0.67)	0.35
Absolute difference from baseline in 2,3-dinor-8-*iso*-PGF_{2a} (ng/mL) †		0.04 (-0.11,0.22)	
5-iPF_{2a}-VI (ng/mL)	1.17 (0.72,1.90)	1.02 (0.60,1.72)	0.42
Absolute difference from baseline in 5-iPF_{2a}-VI (ng/mL) †		-0.05 (-0.42,0.33)	
8,12-iso-iPF_{2a}-VI (ng/mL)	2.96 (2.14,4.50)	2.60 (1.98,4.89)	0.86

Absolute difference from baseline in 8,12-iso-iPF$_{2\alpha}$-VI (ng/mL) †		-0.16 (-0.89,0.68)	
**CysLT**			
**CysLT; Pathway Normalised***	0.07 (-0.24,0.49)	0.10 (-0.33,0.57)	0.99
Difference from baseline in CysLT; Pathway Normalised		0.00 (-0.31,0.34)	
LTE_4 (ng/mL)	0.05 (0.03,0.10)	0.05 (0.03,0.11)	0.80
Absolute difference from baseline in LTE_4 (ng/mL) †		0.00 (-0.03,0.03)	

*Samples taken from scheduled study visits. Excluded any visits where participants were on OCS treatment. Values presented as percentages (%) Means (SD) Median (IQR). *Pathway Normalised: calculated mean of z-scores from analytes of the same pathway using log2-transformed concentrations of each individual analyte. †Absolute differences from baseline: The values reported are the differences in the urinary eicosanoid concentrations on lower and higher dose ICS treatment. Abbreviations: blood eosinophil count (BEC), fractional exhaled nitric-oxide (FeNO), forced expiratory volume in 1 second (FEV_1), forced vital capacity (FVC), asthma control quesionnaire-7 (ACQ-7), body mass index (BMI), prostaglandin-E_2 (PGE_2), prostaglandin-D_2 (PGD_2), prostaglandin-$F_{2\alpha}$ ($PGF_{2\alpha}$), thromboxane (TXA_2), cysteinyl-leukotriene (CysLT)*

Table S3: Demographic, T2-biomarkers and urinary eicosanoid concentrations in participants receiving a Leukotriene receptor antagonist versus those not receiving a Leukotriene receptor antagonist (LTRA)

	No LTRA	LTRA	P-value
**Number of Patients**	147	142	
**Age At Inclusion**	56.0 (13.8)	55.8 (12.3)	0.90
**Gender**			0.56
Female	97 (66.0%)	89 (62.7%)	
Male	50 (34.0%)	53 (37.3%)	
**BMI (kg/m2)**	31.1 (7.4)	32.2 (6.7)	0.19
**Smoking Status**			0.36
Never Smoked	107 (72.8%)	110 (77.5%)	
Ex-Smoker	40 (27.2%)	32 (22.5%)	
**% Predicted FEV1**	76.2 (19.3)	74.9 (19.0)	0.57
**FEV1/FVC**	0.65 (0.11)	0.66 (0.12)	0.59
**FeNO (ppb)**	23 (13,30)	19 (13,28)	0.14
**BEC (x10^9 cells/L)**	0.21 (0.12,0.34)	0.20 (0.10,0.33)	0.52
**PGE_2**			
PGE_2; Pathway Normalised*	0.08 (-0.65,0.65)	-0.07 (-0.75,0.53)	0.28
TetranorPGEM (ng/mL)	21.56 (10.57,37.37)	18.53 (9.56,33.39)	0.28
**PGD_2**			
PGD_2; Pathway Normalised*	-0.12 (-0.56,0.59)	-0.07 (-0.64,0.42)	0.55
2,3-dinor-11β-$PGF_{2\alpha}$ (ng/mL)	0.03 (0.00,0.15)	0.00 (0.00,0.12)	0.39
TetranorPGDM (ng/mL)	2.87 (1.79,3.69)	2.70 (1.64,4.28)	0.90
**$PGF_{2\alpha}$**			
$PGF_{2\alpha}$; Pathway Normalised*	0.05 (-0.30,0.43)	0.03 (-0.57,0.58)	0.83
$PGF_{2\alpha}$ (ng/mL)	1.76 (1.11,3.03)	1.80 (0.95,3.48)	0.93
TetranorPGFM (ng/mL)	0.98 (0.35,2.42)	0.71 (0.23,1.72)	0.10
13,14-dihydro-15-keto$PGF_{2\alpha}$ (ng/mL)	1.78 (1.28,2.50)	2.02 (1.35,3.03)	0.14
**TXA_2**			
TXA_2; Pathway Normalised*	0.14 (-0.19,0.52)	0.22 (-0.31,0.54)	0.81
11-dehydro-2,3-dinor-TXB_2 (ng/mL)	0.20 (0.08,0.42)	0.23 (0.10,0.52)	0.28
11-dehydroTXB_2 (ng/mL)	0.76 (0.43,1.18)	0.68 (0.39,1.10)	0.38
2,3-dinor-TXB_2 (ng/mL)	0.27 (0.16,0.46)	0.29 (0.13,0.53)	0.90
**Isoprostanes**			
Isoprostanes; Pathway Normalised*	0.01 (-0.50,0.51)	0.07 (-0.43,0.48)	0.64
8-iso$PGF_{2\alpha}$ (ng/mL)	0.11 (0.04,0.23)	0.12 (0.06,0.24)	0.55
2,3-dinor-8-iso$PGF_{2\alpha}$ (ng/mL)	0.43 (0.18,0.77)	0.37 (0.16,0.87)	0.79
5-i$PF_{2\alpha}$-VI (ng/mL)	1.13 (0.80,1.73)	1.20 (0.78,1.78)	0.99
8,12-iso-i$PF_{2\alpha}$-VI (ng/mL)	2.87 (1.95,4.03)	3.03 (2.02,4.75)	0.43
**CysLT**			
CysLT; Pathway Normalised*	0.25 (-0.09,0.55)	0.29 (-0.08,0.72)	0.20
LTE_4 (ng/mL)	0.07 (0.04,0.11)	0.07 (0.04,0.14)	0.20

*Data relates to baseline (scheduled) study visits. Values presented as percentages (%) Means (SD) Median (IQR). *Pathway Normalised: calculated mean of z-scores from analytes of the same pathway using log2-transformed concentrations of each individual analyte. Abbreviations: blood eosinophil count (BEC), fractional exhaled nitric-oxide (FeNO), forced expiratory volume in 1 second (FEV_1), forced vital capacity (FVC), asthma control quesionnaire-7 (ACQ-7), body mass index (BMI), prostaglandin-E_2 (PGE_2), prostaglandin-D_2 (PGD_2), prostaglandin-$F_{2\alpha}$ ($PGF_{2\alpha}$), thromboxane (TXA_2), cysteinyl-leukotriene (CysLT).*

Table S4: Sensitivity analysis showing Intra-cross correlation (ICC) between urinary eicosanoids in participants where corticosteroid (CS) treatment was unchanged and who had stable T2-biomarkers (Δ blood eosinophil count [BEC] ≤0.10 x10^9 cells/L, Δ fractional exhaled nitric-oxide [FeNO] ≤10 ppb) and stable symptoms (Δ asthma control questionnaire-7 [ACQ-7] ≤0.5) across scheduled study visits (Visit-1 [Baseline], Visit-3 and Visit-6).

Test	Participants	Observations	ICC (95% CI)
**PGE_2; Pathway Normalised***	63	126	0.64 (0.50,0.78)
TetranorPGEM	63	126	0.64 (0.50,0.78)
**PGD_2; Pathway Normalised***	63	126	0.32 (0.10,0.5)
2,3-dinor-11β-$PGF_{2\alpha}$	63	126	0.28 (0.06,0.51)
TetranorPGDM	63	126	0.41 (0.21,0.61)
**$PGF_{2\alpha}$; Pathway Normalised***	63	126	0.37 (0.17,0.58)
PGF2a	63	126	0.33 (0.12,0.55)
TetranorPGFM	63	126	0.40 (0.19,0.60)
13,14-dihydro-15-keto$PGF_{2\alpha}$	63	126	0.34 (0.13,0.55)
**TXA_2; Pathway Normalised***	63	126	0.39 (0.18,0.59)
11-dehydro-2,3-dinor-TXB_2	63	126	0.49 (0.31,0.67)
11-dehydroTXB_2	63	126	0.16 (0.00,0.49)
2,3-dinor-TXB_2	63	126	0.52 (0.35,0.70)
**Isoprostanes; Pathway Normalised***	63	126	0.57 (0.40,0.73)
8-iso-$PGF_{2\alpha}$	63	126	0.17 (0.00,0.61)
2,3-dinor-8-iso-$PGF_{2\alpha}$	63	126	0.59 (0.42,0.74)
5-$iPF_{2\alpha}$-VI	63	126	0.60 (0.44,0.75)
8,12-iso-$iPF_{2\alpha}$-VI	63	126	0.71 (0.59,0.83)
**CysLT; Pathway Normalised***	63	126	0.26 (0.03,0.49)
LTE_4	63	126	0.26 (0.03,0.49)

**Pathway Normalised: calculated mean of z-scores from analytes of the same pathway using log2-transformed concentrations of each individual analyte. Scheduled study visits were restricted to visits where the participant was on the same CS treatment regimen. Pairwise comparison of visits where Δ ACQ-7 ≤0.5, Δ BEC≤0.10 x10^9 cells/L, Δ FeNO ≤10 ppb. Abbreviations: prostaglandin-E_2 (PGE_2), prostaglandin-D_2 (PGD_2), prostaglandin-$F_{2\alpha}$ ($PGF_{2\alpha}$), thromboxane (TXA_2), cysteinyl-leukotriene (CysLT)*

Table S5: Spearman rank correlation (r) between urinary eicosanoid concentrations and asthma control questionnaire-7 (ACQ-7), fractional exhaled nitric-oxide (FeNO) and blood eosinophil count (BEC) in all participants who were on stable CS treatment at scheduled study visits 1 and 3.

Urine Eicosanoid	BEC (x10^9 cells/L)	FeNO (ppb)	ACQ-7 Score
**PGE_2; Pathway Normalised**	r=0.19 (0.14,0.24)*	r=-0.05 (-0.12,0.02)	r=0.07 (-0.37,0.51)
TetranorPGEM	r=0.19 (0.14,0.24)*	r=-0.05 (-0.12,0.02)	r=0.07 (-0.37,0.51)
**PGD_2; Pathway Normalised**	r=0.20 (-0.01,0.42)*	r=0.03 (-0.16,0.22)	r=0.13 (-0.07,0.33)
2,3-dinor-11β-$PGF_{2α}$	r=0.05 (-0.18,0.28)	r=0.07 (-0.15,0.28)	r=0.07 (-0.07,0.14)
TetranorPGDM	r=0.27 (0.24,0.30)*	r=-0.03 (-0.06,-0.01)	r=0.13 (-0.19,0.44)
**$PGF_{2α}$; Pathway Normalised**	r=0.15 (0.09,0.21)*	r=-0.02 (-0.24,0.20)	r=0.07 (0.05,0.09)
$PGF_{2α}$	r=0.23 (0.15,0.31)*	r=-0.02 (-0.09,0.05)	r=0.04 (-0.12,0.19)
TetranorPGFM	r=0.11 (-0.10,0.31)	r=0.025 (-0.15,0.20)	r=0.09 (0.02,0.17)
13,14-dihydro-15-keto$PGF_{2α}$	r=0.01 (-0.001,0.02)	r=-0.06 (-0.27,0.16)	r=0.05 (-0.12,0.21)
**TXA_2; Pathway Normalised**	r=0.22 (0.21,0.22)*	r=-0.01 (-0.11,0.09)	r=0.13 (-0.13,0.38)
11-dehydro-2,3-dinor-TXB_2	r=0.12 (0.09,0.16)	r=-0.05 (-0.17,0.06)	r=0.06 (-0.23,0.34)
11-dehydroTXB_2	r=0.27 (0.23,0.31)*	r=0.04 (-0.16,0.23)	r=0.14 (-0.19,0.46)*
2,3-dinor-TXB_2	r=0.13 (0.12,0.14)	r=0.03 (0.01,0.05)	r=0.20 (0.14,0.27)*
**Isoprostanes; Pathway Normalised**	r=0.05 (-0.06,0.15)	r=-0.12 (-0.20,-0.04)	r=0.08 (0.06,0.10)
8-iso-$PGF_{2α}$	r=0.07 (-0.23,0.36)	r=-0.01 (-0.17,0.15)	r=0.04 (-0.05,0.13)
2,3-dinor-8-iso-$PGF_{2α}$	r=0.10 (-0.04 ,0.24)	r=-0.06 (-0.23,0.11)	r=0.06 (-0.21,0.34)
5-i$PF_{2α}$-VI	r=0.05 (-0.05,0.15)	r=-0.07 (-0.10,-0.04)	r=0.11 (0.01,0.22)
8,12-iso-i$PF_{2α}$-VI	r=-0.001 (-0.12,0.12)	r=-0.13 (-0.30,0.04)	r=0.02 (-0.105,0.15)
**CysLT; Pathway Normalised**	r=0.20 (0.11,0.30)*	r=-0.04 (-0.05,-0.02)	r=0.15 (0.14,0.17)*
LTE_4	r=0.20 (0.11,0.30)*	r=-0.04 (-0.05,-0.03)	r=0.15 (0.14,0.17)*

*Correlations based on at least 214 observations * P<0.05 (Confidence intervals calculated for each value). Abbreviations: prostaglandin-E_2 (PGE_2), prostaglandin-D_2 (PGD_2), prostaglandin-$F_{2α}$ ($PGF_{2α}$), thromboxane (TXA_2), cysteinyl-leukotriene (CysLT)*

Table S6: Demographics, biomarkers and urinary eicosanoid concentrations in "symptom-high (ACQ-7 ≤1.5)" versus "symptom-low (ACQ-7 >1.5)" participants in the entire study cohort

	Symptom-low	Symptom-high	P-value
**Number of participants**	115	183	
**Gender**			0.004
Female	63 (54.8%)	130 (71.0%)	
Male	52 (45.2%)	53 (29.0%)	
**baseline BMI (kg/m2)**	29.0 (6.2)	33.3 (7.3)	<0.0001
**baseline FEV_1/FVC**	0.66 (0.11)	0.65 (0.12)	0.31
**% Predicted FEV_1**	83.5 (17.5)	70.6 (18.6)	<0.0001
**BEC (x10^9 cells/L)**	0.24 (0.11,0.37)	0.19 (0.10,0.31)	0.07
**FeNO (ppb)**	22 (14,29)	19 (13,28)	0.28
**Periostin (ng/mL)**	54.7 (17.8)	51.4 (15.1)	0.09
**ACQ-7 Score**	0.8 (0.4)	2.7 (0.8)	<0.0001
**PGE_2**			
PGE_2; Pathway Normalised*	-0.05 (-0.79,0.65)	0.04 (-0.57,0.59)	0.62
TetranorPGEM (ng/mL)	18.91 (9.26,37.37)	20.77 (11.46,35.14)	0.62
**PGD_2**			
PGD_2; Pathway Normalised*	-0.16 (-0.70,0.57)	-0.01 (-0.54,0.54)	0.32
2,3-dinor-11β-$PGF_{2α}$ (ng/mL)	0.02 (0.00,0.16)	0.00 (0.00,0.11)	0.18
TetranorPGDM (ng/mL)	2.45 (1.49,3.69)	2.94 (1.97,4.21)	0.008
**$PGF_{2α}$**			
$PGF_{2α}$; Pathway Normalised*	0.10 (-0.37,0.46)	0.03 (-0.41,0.52)	0.88
$PGF_{2α}$ (ng/mL)	1.71 (0.98,2.91)	1.93 (1.16,3.48)	0.23
TetranorPGFM (ng/mL)	1.06 (0.35,2.31)	0.77 (0.25,2.03)	0.19
13,14-dihydro-15-keto$PGF_{2α}$ (ng/mL)	1.63 (1.22,2.67)	2.05 (1.40,3.00)	0.01
**TXA_2**			
TXA_2; Pathway Normalised*	0.15 (-0.31,0.54)	0.26 (-0.20,0.59)	0.25
11-dehydro-2,3-dinor-TXB_2 (ng/mL)	0.21 (0.09,0.45)	0.22 (0.09,0.57)	0.44
11-dehydroTXB_2 (ng/mL)	0.69 (0.38,1.19)	0.77 (0.50,1.17)	0.28
2,3-dinor-TXB_2 (ng/mL)	0.27 (0.14,0.46)	0.29 (0.16,0.54)	0.35
**Isoprostanes**			
Isoprostanes; Pathway Normalised*	-0.02 (-0.65,0.38)	0.08 (-0.28,0.55)	0.01
8-iso-$PGF_{2α}$ (ng/mL)	0.09 (0.02,0.18)	0.14 (0.07,0.25)	0.002
2,3-dinor-8-iso-$PGF_{2α}$ (ng/mL)	0.42 (0.15,0.73)	0.40 (0.18,0.89)	0.61
5-$iPF_{2α}$-VI (ng/mL)	1.06 (0.75,1.57)	1.25 (0.82,1.85)	0.05
8,12-iso-$iPF_{2α}$-VI (ng/mL)	2.58 (1.80,3.91)	3.18 (2.30,4.73)	0.008
**CysLT**			
CysLT; Pathway Normalised*	0.31 (-0.02,0.70)	0.26 (-0.13,0.63)	0.39
LTE_4 (ng/mL)	0.07 (0.04,0.14)	0.07 (0.04,0.12)	0.39

*Samples taken from scheduled study visits. Values presented as percentages (%) Means (SD) Median (IQR). *Pathway Normalised: calculated mean of z-scores from analytes of the same pathway using log2-transformed concentrations of each individual analyte. Definitions: symptom-low = ACQ-7≤1.5; symptom-high = ACQ-7>1.5. Abbreviations: blood eosinophil count (BEC), fractional exhaled nitric-oxide (FeNO), forced expiratory volume in 1 second (FEV_1), forced vital capacity (FVC), asthma control quesionnaire-7 (ACQ-7), body mass index (BMI), prostaglandin-E_2 (PGE_2), prostaglandin-D_2 (PGD_2), prostaglandin-$F_{2α}$ ($PGF_{2α}$), thromboxane (TXA_2), cysteinyl-leukotriene (CysLT)*

Table S7: Demographics, biomarkers and urinary eicosanoid concentrations in "symptom-high (ACQ-7 ≤1.5)" versus "symptom-low (ACQ-7 >1.5)" participants with BMI ≥30kg/m^2 (obese)

	Symptom-low	Symptom-high	P-value
**Number of participants**	38	122	
**Gender**			0.03
Female	23 (60.5%)	95 (77.9%)	
Male	15 (39.5%)	27 (22.1%)	
**baseline BMI (kg/m2)**	35.1 (6.2)	36.7 (6.4)	0.16
**baseline FEV_1/FVC**	0.67 (0.11)	0.67 (0.11)	0.95
**% Predicted FEV_1**	81.6 (17.7)	71.7 (17.3)	0.003
**BEC (x10^9 cells/L)**	0.22 (0.11,0.31)	0.19 (0.10,0.30)	0.54
**FeNO (ppb)**	18 (12,27)	18 (13,27)	0.46
**Periostin (ng/mL)**	47.6 (14.2)	49.3 (14.7)	0.52
**ACQ-7 Score**	0.9 (0.4)	2.8 (0.9)	<0.0001
**PGE_2**			
PGE_2; Pathway Normalised*	0.07 (-0.75,0.67)	-0.06 (-0.57,0.53)	0.96
TetranorPGEM (ng/mL)	21.35 (9.58,38.03)	18.68 (11.46,33.39)	0.96
**PGD_2**			
PGD_2; Pathway Normalised*	-0.06 (-0.51,0.61)	-0.08 (-0.59,0.54)	0.90
2,3-dinor-11β-PGF2α (ng/mL)	0.03 (0.00,0.12)	0.00 (0.00,0.09)	0.18
TetranorPGDM (ng/mL)	2.86 (1.60,3.95)	3.06 (2.02,4.28)	0.18
**$PGF_{2\alpha}$**			
$PGF_{2\alpha}$; Pathway Normalised*	0.09 (-0.51,0.51)	0.05 (-0.36,0.52)	0.90
$PGF_{2\alpha}$ (ng/mL)	2.10 (1.20,3.14)	1.97 (1.25,3.54)	0.84
TetranorPGFM (ng/mL)	1.13 (0.35,2.19)	0.66 (0.21,1.77)	0.19
13,14-dihydro-15-keto$PGF_{2\alpha}$ (ng/mL)	1.63 (1.08,2.73)	2.04 (1.48,3.01)	0.13
**TXA_2**			
TXA_2; Pathway Normalised*	0.05 (-0.24,0.49)	0.26 (-0.20,0.51)	0.32
11-dehydro-2,3-dinor-TXB_2 (ng/mL)	0.26 (0.14,0.42)	0.23 (0.09,0.53)	0.98
11-dehydroTXB_2 (ng/mL)	0.54 (0.29,1.07)	0.74 (0.47,1.16)	0.08
2,3-dinor-TXB_2 (ng/mL)	0.25 (0.14,0.38)	0.29 (0.17,0.53)	0.17
**Isoprostanes**			
Isoprostanes; Pathway Normalised*	0.08 (-0.45,0.51)	0.08 (-0.27,0.53)	0.41
8-iso-$PGF_{2\alpha}$ (ng/mL)	0.08 (0.04,0.15)	0.14 (0.06,0.24)	0.04
2,3-dinor-8-iso-$PGF_{2\alpha}$ (ng/mL)	0.54 (0.26,0.77)	0.35 (0.20,0.89)	0.86
5-i$PF_{2\alpha}$-VI (ng/mL)	1.18 (0.79,1.72)	1.24 (0.79,1.78)	0.55
8,12-iso-i$PF_{2\alpha}$-VI (ng/mL)	2.55 (1.75,3.95)	3.25 (2.39,4.75)	0.08
**CysLT**			
CysLT; Pathway Normalised*	0.31 (-0.08,0.55)	0.24 (-0.08,0.62)	0.99
LTE_4 (ng/mL)	0.07 (0.04,0.11)	0.07 (0.04,0.12)	0.99

*Samples taken from scheduled study visits. Values presented as percentages (%) Means (SD) Median (IQR). *Pathway Normalised: calculated mean of z-scores from analytes of the same pathway using log2-transformed concentrations of each individual analyte. Definitions: Symptom-low = ACQ-7≤1.5; Symptom-high = ACQ-7>1.5;* Obese = BMI ≥30kg/m^2. *Values presented as percentages (%) Means (SD) Median (IQR). Abbreviations: blood eosinophil count (BEC), fractional exhaled nitric-oxide (FeNO), forced expiratory volume in 1 second (FEV_1), forced vital capacity (FVC), asthma control quesionnaire-7 (ACQ-7), body mass index (BMI), prostaglandin-E_2 (PGE_2), prostaglandin-D_2 (PGD_2), prostaglandin-$F_{2\alpha}$ ($PGF_{2\alpha}$), thromboxane (TXA_2), cysteinyl-leukotriene (CysLT)*

Table S8: Demographics, T2-biomarkers and urinary eicosanoid concentrations in "T2-high (fractional exhaled nitric-oxide [FeNO] ≥20 ppb AND blood eosinophil count [BEC] ≥0.15 x10^9 cells/L)" versus "T2-low (FeNO <20 ppb AND BEC <0.15 x10^9 cells/L)" participants in the entire study

	T2-low	T2-high	P-value
**Number of participants**	83	161	
**Gender**			0.06
Female	59 (71.1%)	95 (59.0%)	
Male	24 (28.9%)	66 (41.0%)	
**baseline BMI (kg/m2)**	32.4 (6.6)	31.0 (6.8)	0.11
**baseline FEV_1/FVC**	0.68 (0.12)	0.65 (0.11)	0.02
**% Predicted FEV_1**	77.7 (20.0)	75.2 (18.4)	0.34
**BEC (x10^9 cells/L)**	0.08 (0.04,0.12)	0.31 (0.22,0.51)	<0.0001
**FeNO (ppb)**	13 (10,17)	28 (24,37)	<0.0001
**Periostin (ng/mL)**	47.2 (13.5)	57.3 (17.2)	<0.0001
**ACQ-7 Score**	2.1 (1.1)	1.9 (1.1)	0.20
**PGE_2**			
PGE_2; Pathway Normalised*	-0.05 (-0.74,0.48)	-0.09 (-0.71,0.67)	0.52
TetranorPGEM (ng/mL)	18.91 (9.72,31.67)	18.25 (9.96,38.03)	0.52
**PGD_2**			
PGD_2; Pathway Normalised*	-0.18 (-0.59,0.23)	0.00 (-0.52,0.59)	0.08
2,3-dinor-11β-$PGF_{2\alpha}$ (ng/mL)	0.00 (0.00,0.08)	0.05 (0.00,0.14)	0.05
TetranorPGDM (ng/mL)	2.49 (1.64,3.87)	2.73 (1.79,3.82)	0.52
**$PGF_{2\alpha}$**			
$PGF_{2\alpha}$; Pathway Normalised*	0.02 (-0.34,0.56)	0.05 (-0.41,0.43)	0.64
$PGF_{2\alpha}$ (ng/mL)	1.69 (1.20,3.06)	1.90 (1.11,3.29)	0.76
TetranorPGFM (ng/mL)	0.67 (0.19,2.24)	0.81 (0.30,2.14)	0.88
13,14-dihydro-15-keto$PGF_{2\alpha}$ (ng/mL)	2.11 (1.41,3.11)	1.76 (1.30,2.49)	0.02
**TXA_2**			
TXA2; Pathway Normalised*	0.12 (-0.37,0.48)	0.19 (-0.19,0.54)	0.47
11-dehydro-2,3-dinor-TXB_2 (ng/mL)	0.19 (0.08,0.55)	0.24 (0.09,0.47)	0.92
11-dehydroTXB_2 (ng/mL)	0.63 (0.38,1.04)	0.76 (0.45,1.13)	0.14
2,3-dinor-TXB_2 (ng/mL)	0.27 (0.15,0.48)	0.28 (0.15,0.46)	0.76
**Isoprostanes**			
Isoprostanes; Pathway Normalised*	0.08 (-0.35,0.48)	-0.03 (-0.52,0.40)	0.16
8-iso-$PGF_{2\alpha}$ (ng/mL)	0.12 (0.04,0.24)	0.10 (0.03,0.21)	0.57
2,3-dinor-8-iso-$PGF_{2\alpha}$ (ng/mL)	0.44 (0.18,0.85)	0.36 (0.16,0.74)	0.36
5-i$PF_{2\alpha}$-VI (ng/mL)	1.30 (0.83,1.87)	1.08 (0.79,1.53)	0.02
8,12-iso-i$PF_{2\alpha}$-VI (ng/mL)	3.12 (2.08,4.91)	2.75 (1.93,3.94)	0.13
**CysLT**			
CysLT; Pathway Normalised*	0.09 (-0.49,0.46)	0.34 (-0.00,0.76)	0.0007
LTE_4 (ng/mL)	0.05 (0.02,0.09)	0.08 (0.04,0.15)	0.0007

*Samples taken from scheduled study visits. Values presented as percentages (%) Means (SD) Median (IQR). *Pathway Normalised: calculated mean of z-scores from analytes of the same pathway using log2-transformed concentrations of each individual analyte. Definitions: T2-low = FeNO<20 AND BEC<0.15 x10^9 cells/L); T2-high = FeNO ≥20 ppb AND BEC ≥0.15 x10^9 cells/L. Abbreviations: blood eosinophil count (BEC), fractional exhaled nitric-oxide (FeNO), forced expiratory volume in 1 second (FEV_1), forced vital capacity (FVC), asthma control quesionnaire-7 (ACQ-7), body mass index (BMI), prostaglandin-E_2 (PGE_2), prostaglandin-D_2 (PGD_2), prostaglandin-$F_{2\alpha}$ ($PGF_{2\alpha}$), thromboxane (TXA_2), cysteinyl-leukotriene (CysLT)*

Table S9: Demographics, T2-biomarkers and urinary eicosanoid concentrations in "T2-high (fractional exhaled nitric-oxide [FeNO] ≥20 ppb AND blood eosinophil count [BEC] ≥0.15x10^9cells/L)" versus "T2-low (FeNO <20 ppb AND BEC <0.15 x10^9 cells/L)" participants with BMI ≥30kg/m^2 (obese)

	T2-low	T2-high	P-value
**Number of participants**	48	66	
**Gender**			0.14
Female	38 (79.2%)	44 (66.7%)	
Male	10 (20.8%)	22 (33.3%)	
**baseline BMI (kg/m2)**	36.0 (4.8)	36.4 (6.7)	0.72
**baseline FEV_1/FVC**	0.68 (0.11)	0.67 (0.10)	0.46
**% Predicted FEV_1**	74.3 (17.5)	74.2 (17.8)	0.98
**BEC (x10^9 cells/L)**	0.08 (0.04,0.11)	0.30 (0.21,0.43)	<0.0001
**FeNO (ppb)**	12 (10,17)	28 (24,38)	<0.0001
**Periostin (ng/mL)**	45.4 (12.1)	53.0 (16.1)	0.007
**ACQ-7 Score**	2.3 (1.1)	2.4 (1.1)	0.55
**PGE_2**			
PGE_2; Pathway Normalised*	0.13 (-0.50,0.43)	-0.16 (-0.65,0.53)	0.51
TetranorPGEM (ng/mL)	22.52 (12.27,30.13)	17.05 (10.55,33.39)	0.51
**PGD_2**			
PGD_2; Pathway Normalised*	-0.06 (-0.37,0.38)	-0.09 (-0.41,0.56)	0.81
2,3-dinor-11β-PGF_{2a} (ng/mL)	0.00 (0.00,0.11)	0.02 (0.00,0.13)	0.53
TetranorPGDM (ng/mL)	3.10 (2.01,3.98)	2.82 (1.79,3.98)	0.58
**PGF2α**			
$PGF_{2\alpha}$; Pathway Normalised*	0.12 (-0.21,0.61)	0.05 (-0.50,0.40)	0.26
PGF_{2a} (ng/mL)	2.08 (1.27,3.67)	2.16 (1.35,3.49)	0.92
TetranorPGFM (ng/mL)	0.79 (0.26,1.98)	0.60 (0.21,1.70)	0.51
13,14-dihydro-15-ketoPGF_{2a} (ng/mL)	2.17 (1.62,3.30)	1.87 (1.38,2.49)	0.02
**TXA2**			
TXA_2; Pathway Normalised*	0.24 (-0.14,0.46)	0.26 (-0.12,0.49)	0.86
11-dehydro-2,3-dinor-TXB_2 (ng/mL)	0.23 (0.13,0.51)	0.28 (0.11,0.53)	0.96
11-dehydroTXB_2 (ng/mL)	0.66 (0.42,0.97)	0.66 (0.48,1.08)	0.29
2,3-dinor-TXB_2 (ng/mL)	0.32 (0.16,0.48)	0.27 (0.16,0.44)	0.57
**Isoprostanes**			
Isoprostanes; Pathway Normalised*	0.13 (-0.27,0.67)	-0.02 (-0.43,0.38)	0.05
8-iso-PGF_{2a} (ng/mL)	0.14 (0.06,0.23)	0.10 (0.03,0.24)	0.36
2,3-dinor-8-iso-PGF_{2a} (ng/mL)	0.55 (0.28,1.02)	0.35 (0.20,0.74)	0.11
5-iPF_{2a}-VI (ng/mL)	1.31 (1.06,1.99)	1.09 (0.78,1.53)	0.02
8,12-iso-iPF_{2a}-VI (ng/mL)	3.31 (2.28,5.03)	3.18 (2.17,4.03)	0.39
**CysLT**			
CysLT; Pathway Normalised*	0.15 (-0.27,0.47)	0.33 (0.04,0.65)	0.07
LTE_4 (ng/mL)	0.06 (0.03,0.09)	0.08 (0.05,0.13)	0.07

*Samples taken from scheduled study visits. Values presented as percentages (%) Means (SD) Median (IQR). *Pathway Normalised: calculated mean of z-scores from analytes of the same pathway using log2-transformed concentrations of each individual analyte. Definitions: T2-low = FeNO<20 ppb AND BEC <0.15 x10^9 cells/L; T2-high = FeNO≥20 ppb AND BEC≥0.15 x 10^9cells/L;* Obese = BMI ≥30kg/m^2. *Abbreviations: blood eosinophil count (BEC), fractional exhaled nitric-oxide (FeNO), forced expiratory volume in 1 second (FEV_1), forced vital capacity (FVC), asthma control quesionnaire-7 (ACQ-7), body mass index (BMI), prostaglandin-E_2 (PGE_2), prostaglandin-D_2 (PGD_2), prostaglandin-$F_{2\alpha}$ ($PGF_{2\alpha}$), thromboxane (TXA_2), cysteinyl-leukotriene (CysLT)*

Table S10: Demographics, T2-biomarkers and urinary eicosanoid concentrations in "symptom-high (ACQ-7>1.5)" versus "symptom-low (ACQ-7≤1.5)" participants with T2-low status (fractional exhaled nitric-oxide [FeNO] <20 ppb AND blood eosinophil count [BEC] <0.15 x10^9cells/L) with BMI ≥30kg/m^2 (obese)

	Symptom-low	Symptom-high	P-value
**Number of participants**	13	37	
**Gender**			0.75
Female	10 (76.9%)	30 (81.1%)	
Male	3 (23.1%)	7 (18.9%)	
**baseline BMI (kg/m2)**	35.2 (3.5)	36.3 (5.1)	0.48
**baseline FEV_1/FVC**	0.71 (0.10)	0.68 (0.11)	0.27
**% Predicted FEV_1**	88.0 (17.2)	70.6 (16.1)	0.002
**BEC (x10^9 cells/L)**	0.11 (0.10,0.13)	0.06 (0.03,0.10)	0.007
**FeNO (ppb)**	14 (12,18)	12 (10,17)	0.11
**Periostin (ng/mL)**	44.1 (13.6)	46.3 (11.6)	0.56
**ACQ-7 Score**	0.9 (0.4)	2.7 (0.8)	<0.0001
**PGE2**			
PGE_2; Pathway Normalised*	0.18 (-0.67,0.35)	0.12 (-0.48,0.47)	0.59
TetranorPGEM (ng/mL)	23.80 (10.38,27.89)	22.44 (12.52,31.37)	0.59
**PGD_2**			
PGD_2; Pathway Normalised*	-0.04 (-0.28,0.15)	0.07 (-0.36,0.43)	0.83
2,3-dinor-11β-$PGF_{2\alpha}$ (ng/mL)	0.02 (0.00,0.11)	0.00 (0.00,0.10)	0.61
TetranorPGDM (ng/mL)	2.78 (1.89,4.17)	3.20 (2.04,3.96)	0.51
**$PGF_{2\alpha}$**			
$PGF_{2\alpha}$; Pathway Normalised*	0.13 (-0.21,0.51)	0.10 (-0.20,0.64)	0.94
$PGF_{2\alpha}$ (ng/mL)	2.10 (1.69,3.03)	2.07 (1.34,3.62)	0.90
TetranorPGFM (ng/mL)	1.18 (0.37,1.95)	0.76 (0.18,1.92)	0.51
13,14-dihydro-15-keto$PGF_{2\alpha}$ (ng/mL)	1.63 (1.22,2.47)	2.30 (1.87,3.52)	0.05
**TXA2**			
TXA_2; Pathway Normalised*	-0.04 (-0.24,0.24)	0.32 (-0.04,0.48)	0.12
11-dehydro-2,3-dinor-TXB_2 (ng/mL)	0.20 (0.12,0.31)	0.22 (0.12,0.52)	0.60
11-dehydroTXB_2 (ng/mL)	0.50 (0.39,0.60)	0.76 (0.53,0.98)	0.12
2,3-dinor-TXB_2 (ng/mL)	0.16 (0.14,0.33)	0.32 (0.20,0.50)	0.03
**Isoprostanes**			
Isoprostanes; Pathway Normalised*	0.01 (-0.75,0.25)	0.24 (0.02,0.71)	0.06
8-iso-$PGF_{2\alpha}$ (ng/mL)	0.08 (0.04,0.15)	0.15 (0.10,0.24)	0.07
2,3-dinor-8-iso-$PGF_{2\alpha}$ (ng/mL)	0.56 (0.20,0.60)	0.54 (0.28,1.07)	0.33
5-i$PF_{2\alpha}$-VI (ng/mL)	1.04 (0.66,1.96)	1.42 (1.17,2.02)	0.21
8,12-iso-i$PF_{2\alpha}$-VI (ng/mL)	2.10 (1.76,2.74)	3.73 (2.93,5.38)	0.02
**CysLT**			
CysLT; Pathway Normalised*	-0.02 (-0.33,0.53)	0.17 (-0.20,0.45)	0.82
LTE_4 (ng/mL)	0.04 (0.03,0.10)	0.06 (0.03,0.09)	0.82

*Samples taken from scheduled study visits. Values presented as percentages (%) Means (SD) Median (IQR). *Pathway Normalised: calculated mean of z-scores from analytes of the same pathway using log2-transformed concentrations of each individual analyte. Restricted to participants who were T2-low (FeNO <20 ppb AND BEC<0.15 x10^9 cells/L). Definitions: Symptom-low = ACQ-7≤1.5; Symptom-high = ACQ-7>1.5;* Obese = BMI ≥30kg/m^2. *Abbreviations: blood eosinophil count (BEC), fractional exhaled nitric-oxide (FeNO), forced expiratory volume in 1 second (FEV_1), forced vital capacity (FVC), asthma control quesionnaire-7 (ACQ-7), body mass index (BMI), prostaglandin-E_2 (PGE_2), prostaglandin-D_2 (PGD_2), prostaglandin-$F_{2\alpha}$ ($PGF_{2\alpha}$), thromboxane (TXA_2), cysteinyl-leukotriene (CysLT)*

Table S11: Demographics, T2-biomarkers and urinary eicosanoid concentrations in "symptom-low (ACQ-7≤1.5)" versus "symptom-high (ACQ-7>1.5)" participants with T2-high status (fractional exhaled nitric-oxide [FeNO] ≥20 ppb AND blood eosinophil count [BEC] ≥0.15 x10^9 cells/L)

	Symptom-low	Symptom-high	P-value
**Number of participants**	71	104	
**Gender**			0.01
Female	35 (49.3%)	71 (68.3%)	
Male	36 (50.7%)	33 (31.7%)	
**baseline BMI (kg/m2)**	28.8 (5.9)	32.9 (7.3)	<0.0001
**baseline FEV_1/FVC**	0.66 (0.10)	0.64 (0.12)	0.45
**% Predicted FEV_1**	82.3 (15.0)	69.5 (19.1)	<0.0001
**BEC (x10^9 cells/L)**	0.31 (0.22,0.54)	0.30 (0.21,0.47)	0.41
**FeNO (ppb)**	28 (23,36)	28 (24,39)	0.60
**Periostin (ng/mL)**	57.3 (18.7)	56.3 (16.5)	0.71
**ACQ-7 Score**	0.8 (0.4)	2.7 (0.8)	<0.0001
**PGE_2**			
PGE2; Pathway Normalised*	-0.09 (-0.85,0.67)	-0.00 (-0.66,0.69)	0.80
TetranorPGEM (ng/mL)	18.25 (8.68,38.03)	19.86 (10.50,38.91)	0.80
**PGD_2**			
PGD_2; Pathway Normalised*	-0.21 (-0.70,0.60)	0.16 (-0.46,0.59)	0.18
2,3-dinor-11β-$PGF_{2\alpha}$ (ng/mL)	0.02 (0.00,0.17)	0.05 (0.00,0.13)	0.79
TetranorPGDM (ng/mL)	2.36 (1.51,3.52)	2.91 (2.07,3.97)	0.02
**$PGF_{2\alpha}$**			
$PGF_{2\alpha}$; Pathway Normalised*	0.05 (-0.57,0.40)	0.07 (-0.45,0.51)	0.56
$PGF_{2\alpha}$ (ng/mL)	1.69 (1.04,2.86)	2.16 (1.16,3.68)	0.11
TetranorPGFM (ng/mL)	0.92 (0.31,2.54)	0.73 (0.25,1.96)	0.34
13,14-dihydro-15-keto$PGF_{2\alpha}$ (ng/mL)	1.61 (1.06,2.14)	2.00 (1.37,2.70)	0.02
**TXA_2**			
TXA2; Pathway Normalised*	0.12 (-0.30,0.56)	0.28 (-0.20,0.56)	0.19
11-dehydro-2,3-dinor-TXB_2 (ng/mL)	0.20 (0.08,0.34)	0.26 (0.09,0.54)	0.20
11-dehydroTXB_2 (ng/mL)	0.77 (0.37,1.22)	0.76 (0.49,1.11)	0.92
2,3-dinor-TXB_2 (ng/mL)	0.27 (0.14,0.45)	0.31 (0.16,0.53)	0.36
**Isoprostanes**			
Isoprostanes; Pathway Normalised*	-0.13 (-0.69,0.32)	0.02 (-0.36,0.40)	0.06
8-iso-$PGF_{2\alpha}$ (ng/mL)	0.08 (0.00,0.16)	0.12 (0.06,0.23)	0.03
2,3-dinor-8-iso-$PGF_{2\alpha}$ (ng/mL)	0.36 (0.13,0.73)	0.41 (0.20,0.75)	0.21
5-i$PF_{2\alpha}$-VI (ng/mL)	1.02 (0.78,1.49)	1.11 (0.79,1.63)	0.28
8,12-iso-i$PF_{2\alpha}$-VI (ng/mL)	2.55 (1.80,3.58)	3.12 (2.21,4.18)	0.05
**CysLT**			
CysLT; Pathway Normalised*	0.37 (0.04,0.76)	0.30 (-0.06,0.67)	0.33
LTE_4 (ng/mL)	0.08 (0.05,0.15)	0.07 (0.04,0.13)	0.33

*Samples taken from scheduled study visits. Values presented as percentages (%) Means (SD) Median (IQR). *Pathway Normalised: calculated mean of z-scores from analytes of the same pathway using log2-transformed concentrations of each individual analyte. Restricted to participants who were T2-high (FeNO ≥20 ppb AND BEC ≥0.15 x10^9cells/L). Definitions: Symptom-low = ACQ-7≤1.5; Symptom-high = ACQ-7>1.5. Abbreviations: blood eosinophil count (BEC), fractional exhaled nitric-oxide (FeNO), forced expiratory volume in 1 second (FEV_1), forced vital capacity (FVC), asthma control quesionnaire-7 (ACQ-7), body mass index (BMI), prostaglandin-E_2 (PGE_2), prostaglandin-D_2 (PGD_2), prostaglandin-$F_{2\alpha}$ ($PGF_{2\alpha}$), thromboxane (TXA_2), cysteinyl-leukotriene (CysLT)*

Table S12: Demographics, T2-biomarkers and urinary eicosanoid concentrations in "symptom-low (ACQ-7≤1.5)" versus "symptom-high (ACQ-7>1.5)" participants with T2-high status (fractional exhaled nitric-oxide [FeNO] ≥20 ppb AND blood eosinophil count [BEC] ≥0.15 x10^9 cells/L)" with BMI ≥30kg/m^2 (obese)

	Symptom-low	Symptom-high	P-value
**Number of participants**	15	54	
**Gender**			0.008
Female	6 (40.0%)	41 (75.9%)	
Male	9 (60.0%)	13 (24.1%)	
**baseline BMI (kg/m2)**	36.2 (6.4)	36.4 (6.8)	0.90
**baseline FEV_1/FVC**	0.67 (0.11)	0.67 (0.11)	0.89
**% Predicted FEV_1**	79.4 (15.9)	72.1 (18.2)	0.16
**BEC (x10^9 cells/L)**	0.30 (0.22,0.43)	0.30 (0.20,0.48)	0.89
**FeNO (ppb)**	28 (25,38)	29 (24,42)	0.85
**Periostin (ng/mL)**	47.0 (14.6)	53.9 (16.3)	0.14
**ACQ-7 Score**	1.1 (0.3)	2.8 (0.9)	<0.0001
**PGE_2**			
PGE_2; Pathway Normalised*	-0.09 (-0.71,0.65)	-0.24 (-0.65,0.59)	0.73
TetranorPGEM (ng/mL)	18.25 (10.03,37.37)	15.69 (10.55,35.14)	0.73
**PGD_2**			
PGD_2; Pathway Normalised*	-0.25 (-0.43,0.86)	-0.01 (-0.49,0.43)	0.83
2,3-dinor-11β-$PGF_{2α}$ (ng/mL)	0.02 (0.00,0.27)	0.02 (0.00,0.10)	0.33
TetranorPGDM (ng/mL)	2.73 (1.46,3.94)	2.77 (2.10,4.00)	0.24
**$PGF_{2α}$**			
$PGF_{2α}$; Pathway Normalised*	-0.03 (-0.62,0.22)	0.07 (-0.48,0.43)	0.24
$PGF_{2α}$ (ng/mL)	1.63 (0.70,2.86)	2.21 (1.47,3.57)	0.10
TetranorPGFM (ng/mL)	0.92 (0.22,1.63)	0.53 (0.13,1.70)	0.41
13,14-dihydro-15-keto$PGF_{2α}$ (ng/mL)	1.53 (1.03,1.99)	2.09 (1.48,2.54)	0.04
**TXA_2**			
TXA_2; Pathway Normalised*	0.05 (-0.12,0.52)	0.31 (-0.20,0.49)	0.64
11-dehydro-2,3-dinor-TXB_2 (ng/mL)	0.26 (0.14,0.45)	0.29 (0.09,0.53)	0.88
11-dehydroTXB_2 (ng/mL)	0.62 (0.38,1.40)	0.71 (0.44,1.06)	0.95
2,3-dinor-TXB_2 (ng/mL)	0.25 (0.14,0.35)	0.29 (0.16,0.46)	0.40
**Isoprostanes**			
Isoprostanes; Pathway Normalised*	0.09 (-0.78,0.38)	-0.02 (-0.32,0.34)	0.56
8-iso-$PGF_{2α}$ (ng/mL)	0.07 (0.02,0.14)	0.11 (0.03,0.24)	0.31
2,3-dinor-8-iso-$PGF_{2α}$ (ng/mL)	0.37 (0.13,0.73)	0.34 (0.20,0.74)	0.63
5-i$PF_{2α}$-VI (ng/mL)	0.96 (0.70,1.48)	1.11 (0.79,1.53)	0.40
8,12-iso-i$PF_{2α}$-VI (ng/mL)	2.61 (1.66,3.95)	3.33 (2.37,4.62)	0.29
**CysLT**			
*CysLT; Pathway Normalised**	0.32 (0.03,0.55)	0.33 (0.05,0.66)	0.60
LTE_4 (ng/mL)	0.07 (0.05,0.11)	0.08 (0.05,0.13)	0.60

*Samples taken from scheduled study visits. Values presented as percentages (%) Means (SD) Median (IQR). *Pathway Normalised: calculated mean of z-scores from analytes of the same pathway using log2-transformed concentrations of each individual analyte. Restricted to participants who were T2-high (FeNO ≥20 ppb AND BEC≥0.15 x10^9 cells/L). Definitions: Symptom-low = ACQ-7≤1.5; Symptom-high = ACQ-7>1.5;* Obese = BMI ≥30kg/m^2. *Abbreviations: blood eosinophil count (BEC), fractional exhaled nitric-oxide (FeNO), forced expiratory volume in 1 second (FEV_1), forced vital capacity (FVC), asthma control quesionnaire-7 (ACQ-7), body mass index (BMI), prostaglandin-E_2 (PGE_2), prostaglandin-D_2 (PGD_2), prostaglandin-$F_{2α}$ ($PGF_{2α}$), thromboxane (TXA_2), cysteinyl-leukotriene (CysLT)*

Table S13: Demographics, T2-biomarkers and urinary eicosanoid concentrations at baseline and during an exacerbation in the overall study cohort

	Baseline	Exacerbation	P-value
**Number of participants**	70	70	
**Gender**			1.00
Female	49 (70.0%)	49 (70.0%)	
Male	21 (30.0%)	21 (30.0%)	
**baseline BMI (kg/m2)**	34.0 (7.5)	34.0 (7.5)	
**% Predicted FEV_1**	72.6 (19.6)	62.8 (20.0)	<0.0001
**BEC ($x10^9$ cells/L)**	0.21 (0.14,0.33)	0.16 (0.04,0.29)	0.13
**Absolute difference from baseline in BEC ($x10^9$cells/L)**		-0.06 (-0.15,0.11)	
**FeNO (ppb)**	17 (12,28)	25 (10,38)	0.02
**Absolute difference from baseline in FeNO (ppb)**		3 (-5,17)	
**Periostin (ng/mL)**	52.2 (14.6)	53.0 (17.8)	0.66
**Absolute difference from baseline in Periostin (ng/mL)**		-0.2 (-5.0,3.9)	
**ACQ-7 Score**	2.2 (1.2)	3.5 (1.0)	<0.0001
**Absolute difference from baseline in ACQ-7 Score**		1.4 (0.9,1.9)	
**PGE2**			
**PGE_2; Pathway Normalised***	-0.11 (-0.88,0.45)	0.10 (-0.73,0.60)	0.32
Difference from baseline in PGE_2; Pathway Normalised		0.18 (-0.57,0.80)	
TetranorPGEM (ng/mL)	17.89 (8.47,30.78)	21.96 (9.80,35.56)	0.19
Absolute difference from baseline in TetranorPGEM (ng/mL) †		3.52 (-8.22,14.16)	
**PGD_2**			
**PGD_2; Pathway Normalised***	-0.08 (-0.64,0.54)	-0.14 (-0.66,0.47)	0.53
Difference from baseline in PGD_2; Pathway Normalised		0.06 (-0.38,0.59)	
2,3-dinor-11β-PGF_{2a} (ng/mL)	0.02 (0.00,0.14)	0.02 (0.00,0.12)	0.2258
Absolute difference from baseline in 2,3-dinor-11β-PGF_{2a} (ng/mL) †		0.00 (-0.02,0.07)	
TetranorPGDM (ng/mL)	2.49 (1.55,3.95)	2.77 (1.59,3.74)	0.4954
Absolute difference from baseline in TetranorPGDM *(ng/mL)* †		0.23 (-0.97,1.24)	
**$PGF_{2\alpha}$**			

**$PGF_{2\alpha}$; Pathway Normalised***	-0.05 (-0.46,0.43)	0.12 (-0.49,0.50)	0.19
Difference from baseline in $PGF_{2\alpha}$; Pathway Normalised		0.11 (-0.28,0.58)	
PGF_{2a} (ng/mL)	1.93 (1.20,3.55)	2.08 (1.05,3.76)	0.3255
Absolute difference from baseline in *(ng/mL)* †		0.16 (-0.69,1.32)	
TetranorPGFM (ng/mL)	0.55 (0.15,1.63)	1.05 (0.23,2.57)	0.18
Absolute difference from baseline in TetranorPGFM (ng/mL) †		0.10 (-0.34,1.08)	
13,14-dihydro-15-ketoPGF_{2a} (ng/mL)	1.93 (1.34,2.78)	1.87 (1.18,2.49)	0.47
Absolute difference from baseline in 13,14-dihydro-15-ketoPGF_{2a} *(ng/mL)* †		0.14 (-0.41,0.58)	
**TXA_2**			
**TXA_2; Pathway Normalised***	0.23 (-0.29,0.54)	0.31 (-0.18,0.64)	0.10
Difference from baseline in TXA_2; Pathway Normalised		0.15 (-0.29,0.40)	
11-dehydro-2,3-dinor-TXB_2 (ng/mL)	0.22 (0.09,0.46)	0.30 (0.11,0.59)	0.01
Absolute difference from baseline in 11-dehydro-2,3-dinor-TXB_2 *(ng/mL)* †		0.07 (-0.07,0.28)	
11-dehydroTXB_2 (ng/mL)	0.71 (0.38,1.07)	0.77 (0.44,1.35)	0.03
Absolute difference from baseline in 11-dehydroTXB_2 (ng/mL) †		0.16 (-0.24,0.49)	
2,3-dinor-TXB_2 (ng/mL)	0.29 (0.16,0.53)	0.35 (0.19,0.54)	0.11
Absolute difference from baseline in 2,3-dinor-TXB_2 *(ng/mL)* †		0.03 (-0.10,0.25)	
**Isoprostanes**			
**Isoprostanes; Pathway Normalised***	0.08 (-0.34,0.40)	0.02 (-0.41,0.55)	0.20
Difference from baseline in Isoprostanes; Pathway Normalised		0.14 (-0.29,0.54)	
8-iso-PGF_{2a} (ng/mL)	0.13 (0.06,0.24)	0.15 (0.07,0.33)	0.06
Absolute difference from baseline in 8-*iso*-PGF_{2a} (ng/mL) †		0.02 (-0.04,0.13)	
2,3-dinor-8-iso-PGF_{2a} (ng/mL)	0.41 (0.20,0.76)	0.39 (0.16,0.91)	0.36
Absolute difference from baseline in 2,3-dinor-8-*iso*-PGF_{2a} (ng/mL) †		0.02 (-0.25,0.34)	
5-iPF_{2a}-VI (ng/mL)	1.10 (0.72,1.67)	1.15 (0.75,1.82)	0.35
Absolute difference from baseline in 5-iPF_{2a}-VI (ng/mL) †		0.05 (-0.30,0.32)	
8,12-iso-iPF_{2a}-VI (ng/mL)	3.02 (1.84,4.32)	2.90 (1.86,4.45)	0.36
Absolute difference from baseline in 8,12-*iso*-iPF_{2a}-VI *(ng/mL)* †		0.21 (-0.60,0.75)	
**CysLT**			
**CysLT; Pathway Normalised***	0.23 (-0.18,0.60)	0.13 (-0.30,0.47)	0.54

Difference from baseline in CysLT; Pathway Normalised		0.00 (-0.47,0.25)	
LTE_4 (ng/mL)	0.06 (0.03,0.12)	0.05 (0.03,0.10)	0.38
Abs Diff from baseline in LTE_4 (ng/mL) *†*		-0.00 (-0.03,0.02)	

*Exacerbation visit values presented as percentages (%), mean ± standard deviation (SD) or median (IQR). *Pathway Normalised: calculated mean of z-scores from analytes of the same pathway using log2-transformed concentrations of each individual analyte. †Absolute differences from baseline: The values reported are the differences in urinary eicosanoid concentrations during exacerbation from baseline. Abbreviations: blood eosinophil count (BEC), fractional exhaled nitric-oxide (FeNO), forced expiratory volume in 1 second (FEV_1), forced vital capacity (FVC), asthma control quesionnaire-7 (ACQ-7), body mass index (BMI), prostaglandin-E_2 (PGE_2), prostaglandin-D_2 (PGD_2), prostaglandin-$F_{2\alpha}$ ($PGF_{2\alpha}$), thromboxane (TXA_2), cysteinyl-leukotriene (CysLT)*

Table S14: Subgroup analysis of demographics, T2-biomarkers and urinary eicosanoids of participants who were "T2-high (fractional exhaled nitric-oxide [FeNO] ≥20 ppb AND blood eosinophil count [BEC] ≥0.15 x10^9 cells/L)" and "T2-low (FeNO <20 ppb AND BEC <0.15 x10^9cells/L)" during an exacerbation

	T2-low	T2-high	P-value
**Number of participants**	18	29	
**Gender**			0.37
Female	14 (77.8%)	19 (65.5%)	
Male	4 (22.2%)	10 (34.5%)	
**% Predicted FEV_1**	65.8 (23.4)	62.1 (19.6)	0.57
**BEC (x10^9cells/L)**	0.04 (0.02,0.10)	0.32 (0.22,0.54)	<0.0001
Absolute difference from baseline in BEC (x10^9cells/L)	-0.15 (-0.19,-0.08)	0.13 (0.02,0.21)	<0.0001
**FeNO (ppb)**	11 (9,14)	34 (28,47)	<0.0001
Absolute difference from baseline in FeNO (ppb)	-3 (-12,-2)	17 (7,27)	<0.0001
**Periostin (ng/mL)**	49.2 (14.0)	54.9 (23.7)	0.37
Absolute difference from baseline in Periostin (ng/mL)	-2.7 (-5.0,0.8)	0.7 (-5.6,5.6)	0.26
**ACQ-7 Score**	3.4 (1.1)	3.6 (0.9)	0.43
Absolute difference from baseline in ACQ-7 Score	1.3 (0.9,1.9)	1.3 (0.6,1.9)	0.96
**PGE_2**			
**PGE_2; Pathway Normalised***	0.10 (-1.10,1.05)	0.19 (-0.19,0.55)	0.93
Difference from baseline in PGE_2; Pathway Normalised	0.16 (-0.57,0.63)	0.37 (-0.29,1.24)	0.19
TetranorPGEM (ng/mL)	22.70 (6.82,55.25)	23.87 (16.59,33.97)	0.93
Absolute difference from baseline in TetranorPGEM *(ng/mL) †*	3.05 (-7.18,20.11)	8.38 (-3.14,15.71)	0.5286
**PGD_2**			
**PGD_2; Pathway Normalised***	-0.33 (-0.90,0.36)	0.26 (-0.34,0.69)	0.08
Difference from baseline in PGD_2; Pathway Normalised	-0.37 (-0.77,0.14)	0.32 (-0.11,1.01)	0.02
2,3-dinor-11β-$PGF_{2\alpha}$ (ng/mL)	0.00 (0.00,0.11)	0.05 (0.00,0.12)	0.21
Absolute difference from baseline in 2,3-dinor-11β-$PGF_{2\alpha}$ *(ng/mL) †*	0.00 (-0.04,0.01)	0.02 (-0.00,0.10)	0.10
TetranorPGDM (ng/mL)	2.27 (1.28,3.80)	3.23 (2.00,4.86)	0.08
Absolute difference from baseline in TetranorPGDM *(ng/mL) †*	-0.35 (-1.46,0.71)	0.76 (-0.19,1.72)	0.02

**$PGF_{2\alpha}$**			
**$PGF_{2\alpha}$; Pathway Normalised***	0.06 (-0.84,0.50)	0.22 (0.03,0.57)	0.26
Difference from baseline in $PGF_{2\alpha}$; Pathway Normalised	0.09 (-0.25,0.46)	0.25 (-0.11,0.79)	0.24
$PGF_{2\alpha}$	1.44 (1.00,3.58)	2.49 (1.82,4.26)	0.11
Absolute difference from baseline in $PGF_{2\alpha}$	0.23 (-1.19,2.18)	0.51 (-0.31,1.58)	0.70
TetranorPGFM (ng/mL) †	1.02 (0.23,3.28)	1.14 (0.62,1.80)	0.86
Absolute difference from baseline in TetranorPGFM *(ng/mL) †*	0.28 (-0.34,1.71)	0.36 (-0.24,1.06)	0.80
13,14-dihydro-15-keto$PGF_{2\alpha}$ (ng/mL)	1.95 (1.26,2.79)	1.78 (1.19,2.49)	0.81
Absolute difference from baseline in 13,14-dihydro-15-keto$PGF_{2\alpha}$ *(ng/mL) †*	-0.05 (-0.52,0.47)	0.27 (-0.25,0.83)	0.50
**TXA_2**			
**TXA_2; Pathway Normalised***	0.25 (-0.29,0.41)	0.41 (0.08,0.67)	0.17
Difference from baseline in TXA_2; Pathway Normalised	0.24 (-0.36,0.53)	0.22 (0.05,0.62)	0.44
11-dehydro-2,3-dinor-TXB_2 (ng/mL)	0.15 (0.08,0.30)	0.46 (0.22,0.71)	0.03
Absolute difference from baseline in 11-dehydro-2,3-dinor-TXB_2 *(ng/mL) †*	0.06 (-0.12,0.19)	0.11 (0.01,0.41)	0.15
11-dehydroTXB_2 (ng/mL)	0.84 (0.47,1.19)	0.91 (0.41,1.74)	0.57
Absolute difference from baseline in 11-dehydroTXB_2 *(ng/mL) †*	0.23 (-0.36,0.60)	0.25 (-0.00,0.60)	0.64
2,3-dinor-TXB_2 (ng/mL)	0.34 (0.19,0.46)	0.40 (0.25,0.56)	0.34
Absolute difference from baseline in 2,3-dinor-TXB_2 *(ng/mL) †*	-0.00 (-0.13,0.27)	0.06 (-0.01,0.23)	0.38
**Isoprostanes**			
**Isoprostanes; Pathway Normalised***	0.06 (-0.43,0.55)	0.02 (-0.37,0.57)	0.88
Difference from baseline in Isoprostanes; Pathway Normalised	0.06 (-0.30,0.30)	0.27 (-0.02,0.59)	0.09
8-iso-$PGF_{2\alpha}$	0.19 (0.06,0.47)	0.16 (0.07,0.37)	0.79
Absolute difference from baseline in 8-*iso*-$PGF_{2\alpha}$ *(ng/mL) †*	0.06 (-0.03,0.17)	0.04 (-0.00,0.13)	0.68
2,3-dinor-8-iso-$PGF_{2\alpha}$ (ng/mL)	0.44 (0.14,1.54)	0.54 (0.32,0.94)	0.90
Absolute difference from baseline in 2,3-dinor-8-*iso*-$PGF_{2\alpha}$ *†*	0.15 (-0.29,0.99)	0.13 (-0.09,0.37)	0.79
5-i$PF_{2\alpha}$-VI (ng/mL)	1.06 (0.81,1.82)	1.19 (0.86,1.58)	0.89
Absolute difference from baseline in 5-i$PF_{2\alpha}$-VI	-0.08 (-0.52,0.30)	0.23 (-0.02,0.63)	0.10
8,12-iso-i$PF_{2\alpha}$-VI (ng/mL) †	2.81 (2.04,4.89)	2.76 (1.85,4.15)	0.74
Absolute difference from baseline in 8,12-*iso*-i$PF_{2\alpha}$ -VI *(ng/mL) †*	-0.08 (-1.59,0.68)	0.28 (-0.55,0.97)	0.36
**CysLT**			

**CysLT; Pathway Normalised***	0.09 (-2.09,0.24)	0.34 (-0.22,0.54)	0.05
Difference from baseline in CysLT; Pathway Normalised	-0.07 (-0.30,0.00)	0.13 (-0.20,0.55)	0.03
LTE_4 (ng/mL)	0.05 (0.00,0.07)	0.08 (0.03,0.11)	0.05
Absolute difference from baseline in LTE_4 †	-0.01 (-0.05,-0.00)	0.01 (-0.03,0.04)	0.04

*Exacerbation visits values presented as percentages. *Pathway Normalised: calculated mean of z-scores from analytes of the same pathway using log2-transformed concentrations of each individual analyte. †Absolute differences from baseline: The values reported are the differences in urinary eicosanoid concentrations during exacerbation from baseline. Definitions: T2-low exacerbations = FeNO<20 AND BEC <0.15 x10^9 cells/L; T2-high exacerbations = FeNO ≥20 ppb AND BEC ≥0.15 x10^9cells/L. Abbreviations: blood eosinophil count (BEC), fractional exhaled nitric-oxide (FeNO), forced expiratory volume in 1 second (FEV_1), forced vital capacity (FVC), asthma control quesionnaire-7 (ACQ-7), prostaglandin-E_2 (PGE_2), prostaglandin-D_2 (PGD_2), prostaglandin-$F_{2\alpha}$ ($PGF_{2\alpha}$), thromboxane (TXA_2), cysteinyl-leukotriene (CysLT)*

Table S15: Spearman rank correlation (r) between urinary eicosanoid concentrations, blood eosinophil count (BEC), fractional exhaled nitric-oxide (FeNO) and Asthma control questionnaire-7 (ACQ-7) during an exacerbation

Urine Eicosanoid	BEC (x10^9 cells/L)	FeNO (ppb)	ACQ-7 Score
**PGE_2; Pathway Normalised**	r=0.24 (0.18,0.30)*	r=0.09 (-0.13,0.31)	r=0.15 (0.11,0.19)
TetranorPGEM	r=0.24 (0.18,0.30)*	r=0.09 (-0.13,0.31)	r=0.148 (0.11,0.19)
**PGD_2; Pathway Normalised**	r=0.32 (0.22,0.41)*	r=0.03 (-0.09,0.15)	r=0.13 (0.09,0.18)
2,3-dinor-11*β*-$PGF_{2\alpha}$	r=0.11 (0.07,0.15)	r=0.11 (0.006,0.22)	r=0.01 (-0.09,0.12)
TetranorPGDM	r=0.34 (0.16,0.53)*	r=-0.06 (-0.16,0.04)	r=0.18 (0.15,0.20)
**$PGF_{2\alpha}$; Pathway Normalised**	r=0.25 (0.03,0.47)*	r=0.07 (-0.21,0.35)	r=0.12 (0.04,0.20)
$PGF_{2\alpha}$	r=0.22 (0.11,0.33)*	r=0.03 (-0.22,0.28)	r=0.14 (0.08,0.20)
TetranorPGFM	r=0.25 (0.12,0.37)*	r=-0.004 (-0.26,0.25)	r=-0.03 (-0.40,0.34)
13,14-dihydro-15-keto$PGF_{2\alpha}$	r=0.16 (0.11,0.22)	r=0.18 (-0.09,0.44)	r=0.04 (-0.18,0.27)
**TXA_2; Pathway Normalised**	r=0.23 (0.20 ,0.27)*	r=0.13 (0.13,0.13)	r=0.26 (0.24,0.28)*
11-dehydro-2,3-dinor-TXB_2	r=0.21 (0.08,0.35)*	r=0.15 (0.14,0.16)	r=0.19 (0.17,0.21)
11-dehydroTXB_2	r=0.16 (0.11,0.20)	r=0.03 (-0.06,0.12)	r=0.35 (0.35,0.36)†
2,3-dinor-TXB_2	r=0.32 (0.29,0.35)*	r=0.20 (0.19,0.22)†	r=0.22 (0.21,0.22)†
**Isoprostanes; Pathway Normalised**	r=0.22 (0.20,0.25)*	r=0.18 (-0.05,0.41)	r=0.09 (-0.22,0.40)
8-*iso*-$PGF_{2\alpha}$	r=0.05 (0.01,0.08)	r=0.09 (-0.01,0.18)	r=0.21 (-0.02,0.44)†
2,3-dinor-8-*iso*-$PGF_{2\alpha}$	r=0.12 (0.04,0.21)	r=0.05 (-0.06,0.16)	r=0.08 (-0.23,0.39)
5-i$PF_{2\alpha}$-VI	r=0.24 (0.10,0.38)*	r=0.182 (0.06,0.30)	r=0.082 (0.07,0.10)
8,12-*iso*-i$PF_{2\alpha}$-VI	r=0.24 (0.06,0.42)*	r=0.17 (-0.12,0.45)	r=-0.06 (-0.31,0.18)
**CysLT; Pathway Normalised**	r=0.43 (0.37,0.49)*	r=0.27 (-0.13,0.66)*	r=0.24 (0.07,0.41)*
LTE_4	r=0.43 (0.37,0.49)*	r=0.27 (-0.13,0.66)*	r=0.24 (0.07,0.41)*

*Correlations based on at least 98 observations *P<0.05. Abbreviations: blood eosinophil count (BEC) fractional exhaled nitric-oxide (FeNO, asthma control quesionnaire-7 (ACQ-7), prostaglandin-E_2 (PGE_2), prostaglandin-D_2 (PGD_2), prostaglandin-$F_{2\alpha}$ ($PGF_{2\alpha}$), thromboxane (TXA_2), cysteinyl-leukotriene (CysLT)*

Table S16: Sensitivity analysis showing demographics, T2-biomarkers and urinary eicosanoid concentrations in T2-low and T2-high participants having excluded participants on a leukotriene receptor antagonist (LTRA)

	T2-Low	T2-high	P-value
**Number of Patients**	36	87	
**Gender**			0.51
Female	25 (69.4%)	55 (63.2%)	
Male	11 (30.6%)	32 (36.8%)	
**Baseline BMI (kg/m^2)**	32.8 (7.1)	30.1 (6.9)	0.06
**Baseline FEV_1/FVC**	0.67 (0.12)	0.64 (0.11)	0.26
**% Predicted FEV_1**	73.4 (17.6)	75.8 (19.9)	0.55
**BEC (10^9 cells/L)**	0.09 (0.04,0.12)	0.31 (0.22,0.54)	<0.0001
**FeNO (ppb)**	12 (9,15)	30 (24,39)	<0.0001
**ACQ7 Score**	2.2 (1.1)	1.8 (1.1)	0.08
**PGE_2**			
PGE_2; Pathway Normalised	-0.20 (-0.72,0.55)	-0.14 (-0.71,0.70)	0.62
tetranorPGEM (ng/mL)	16.46 (9.91,34.11)	17.43 (9.96,39.35)	0.62
**PGD_2**			
PGD_2; Pathway Normalised	-0.21 (-0.44,0.21)	0.05 (-0.56,0.66)	0.17
2,3-dinor-11-B-$PGF_{2\alpha}$ (ng/mL)	0.00 (0.00,0.06)	0.06 (0.00,0.17)	0.04
TetranorPGDM (ng/mL)	2.85 (1.81,3.66)	2.73 (1.75,3.49)	0.96
**$PGF_{2\alpha}$**			
$PGF_{2\alpha}$; Pathway Normalised	0.05 (-0.30,0.34)	0.05 (-0.32,0.43)	0.94
$PGF_{2\alpha}$ (ng/mL)	1.61 (0.96,2.85)	1.98 (1.11,3.02)	0.53
TetranorPGFM (ng/mL)	0.74 (0.26,2.11)	0.88 (0.33,2.89)	0.76
13,14-dihydro-15-keto$PGF_{2\alpha}$ (ng/mL)	2.10 (1.40,2.91)	1.67 (1.32,2.39)	0.1
**TXA_2**			
TXA_2; Pathway Normalised	-0.04 (-0.76,0.45)	0.15 (-0.17,0.55)	0.19
11-dehydro-2,3-dinor-TXB_2 (ng/mL)	0.16 (0.05,0.54)	0.20 (0.09,0.53)	0.43
11-dehydroTXB_2 (ng/mL)	0.62 (0.35,1.07)	0.83 (0.47,1.18)	0.16
2,3-dinor-TXB_2 (ng/mL)	0.24 (0.14,0.39)	0.28 (0.16,0.46)	0.12
**Isoprostanes**			
Isoprostanes; Pathway Normalised	0.12 (-0.37,0.52)	-0.05 (-0.50,0.43)	0.41
8-iso$PGF_{2\alpha}$ (ng/mL)	0.11 (0.05,0.27)	0.10 (0.03,0.22)	0.46
2,3-dinor-8-iso$PGF_{2\alpha}$ (ng/mL)	0.39 (0.18,0.96)	0.40 (0.16,0.86)	0.82
5-i$PF_{2\alpha}$-VI (ng/mL)	1.25 (1.01,1.76)	1.06 (0.79,1.69)	0.16
8,12-iso-i$PF_{2\alpha}$-VI (ng/mL)	3.21 (2.11,4.62)	2.76 (1.95,3.95)	0.42
**CysLT**			
CysLT; Pathway Normalised	0.09 (-0.30,0.42)	0.32 (-0.06,0.67)	0.05
LTE_4 (ng/mL)	0.05 (0.03,0.09)	0.07 (0.04,0.13)	0.05

*Samples taken from scheduled study visits. Values presented as percentages (%) Means (SD) Median (IQR). *Pathway Normalised: calculated mean of z-scores from analytes of the same pathway using log2-transformed concentrations of each individual analyte. Definitions: T2-low = FeNO<20 AND BEC<0.15 x10^9 cells/L); T2-high = FeNO ≥20 ppb AND BEC ≥0.15 x10^9 cells/L. Abbreviations: blood eosinophil count (BEC), fractional exhaled nitric-oxide (FeNO), forced expiratory volume in 1 second (FEV_1), forced vital capacity (FVC), asthma control quesionnaire-7 (ACQ-7), body mass index (BMI), prostaglandin-E_2 (PGE_2), prostaglandin-D_2 (PGD_2), prostaglandin-$F_{2\alpha}$ ($PGF_{2\alpha}$), thromboxane (TXA_2), cysteinyl-leukotriene (CysLT)*

Table S17: Sensitivity analysis showing demographics, T2-biomarkers and urinary eicosanoid concentrations in T2-low and T2-high participants having excluded participants with aspirin exacerbated respiratory disease (AERD)

	T2-Low	T2-high	P-value
**Number of Patients**	67	138	
**Gender**			0.17
Female	45 (67.2%)	79 (57.2%)	
Male	22 (32.8%)	59 (42.8%)	
**Baseline BMI (kg/m^2)**	32.4 (6.9)	30.7 (6.6)	0.10
**Baseline FEV_1/FVC**	0.68 (0.13)	0.64 (0.11)	0.06
**% Predicted FEV_1**	77.8 (20.4)	75.1 (18.7)	0.34
**BEC (10^9 cells/L)**	0.09 (0.05,0.12)	0.30 (0.22,0.48)	<0.0001
**FeNO (ppb)**	12 (9,17)	28 (23,37)	<0.0001
**ACQ7 Score**	2.1 (1.1)	1.9 (1.2)	0.21
**PGE_2**			
PGE_2; Pathway Normalised	-0.08 (-0.72,0.47)	-0.13 (-0.75,0.67)	0.66
tetranorPGEM (ng/mL)	18.45 (9.89,31.37)	17.61 (9.64,38.03)	0.66
**PGD_2**			
PGD_2; Pathway Normalised	-0.20 (-0.63,0.19)	0.00 (-0.56,0.60)	0.07
2,3-dinor-11-B-$PGF_{2\alpha}$ (ng/mL)	0.00 (0.00,0.06)	0.04 (0.00,0.14)	0.03
TetranorPGDM (ng/mL)	2.49 (1.67,3.67)	2.70 (1.77,3.82)	0.64
**$PGF_{2\alpha}$**			
$PGF_{2\alpha}$; Pathway Normalised	0.08 (-0.28,0.64)	0.04 (-0.52,0.43)	0.18
$PGF_{2\alpha}$ (ng/mL)	1.71 (1.33,3.24)	1.93 (1.09,3.02)	0.66
TetranorPGFM (ng/mL)	0.76 (0.28,2.45)	0.82 (0.28,2.07)	0.71
13,14-dihydro-15-keto$PGF_{2\alpha}$ (ng/mL)	2.23 (1.53,3.12)	1.72 (1.29,2.39)	0.003
**TXA_2**			
TXA_2; Pathway Normalised	0.07 (-0.37,0.45)	0.17 (-0.23,0.53)	0.36
11-dehydro-2,3-dinor-TXB_2 (ng/mL)	0.18 (0.07,0.52)	0.22 (0.09,0.47)	0.75
11-dehydroTXB_2 (ng/mL)	0.62 (0.38,1.00)	0.75 (0.43,1.11)	0.15
2,3-dinor-TXB_2 (ng/mL)	0.27 (0.14,0.41)	0.28 (0.14,0.46)	0.57
**Isoprostanes**			
Isoprostanes; Pathway Normalised	0.13 (-0.30,0.60)	-0.02 (-0.53,0.40)	0.03
8-isoPGF_{2a} (ng/mL)	0.12 (0.07,0.26)	0.10 (0.03,0.21)	0.10
2,3-dinor-8-isoPGF_{2a} (ng/mL)	0.42 (0.18,0.86)	0.36 (0.15,0.74)	0.43
5-iPF2_a-VI (ng/mL)	1.30 (0.83,1.94)	1.07 (0.78,1.59)	0.03
8,12-iso-iPF_{2a}-VI (ng/mL)	3.31 (2.17,5.20)	2.71 (1.92,3.95)	0.04
**CysLT**			
CysLT; Pathway Normalised	0.08 (-0.49,0.45)	0.28 (-0.02,0.70)	0.003
LTE_4 (ng/mL);	0.05 (0.02,0.09)	0.07 (0.04,0.14)	0.003

*Samples taken from scheduled study visits. Values presented as percentages (%) Means (SD) Median (IQR). *Pathway Normalised: calculated mean of z-scores from analytes of the same pathway using log2-transformed concentrations of each individual analyte. Definitions: T2-low = FeNO<20 AND BEC<0.15 x10^9 cells/L); T2-high = FeNO ≥20 ppb AND BEC ≥0.15 x10^9 cells/L. Abbreviations: blood eosinophil count (BEC), fractional exhaled nitric-oxide (FeNO), forced expiratory volume in 1 second (FEV_1), forced vital capacity (FVC), asthma control quesionnaire-7 (ACQ-7), body mass index (BMI), prostaglandin-E_2 (PGE_2), prostaglandin-D_2 (PGD_2), prostaglandin-$F_{2\alpha}$ ($PGF_{2\alpha}$), thromboxane (TXA_2), cysteinyl-leukotriene (CysLT)*

References:

[E1] Hanratty CE, Matthews JG, Arron JR et al. A randomised pragmatic trial of corticosteroid optimization in severe asthma using a composite biomarker algorithm to adjust corticosteroid dose versus standard care: study protocol for a randomised trial. Trials 2018; 19: 5.

[E2] Dweik RA, Boggs PB, Erzurum SC et al. An official ATS clinical practice guideline: Interpretation of exhaled nitric oxide levels (FENO) for clinical applications. American Journal of Respiratory and Critical Care Medicine 2011; 184.

[E3] Wanger J, Clausen JA, Coates A. 'ATS/ERS task force: Standardisation of lung function testing. European Respiratory Journal 2005; 26: 511–522 2005: 511–2.

[E4] Quanjer PH, Stanojevic S, Cole TJ et al. Multi-ethnic reference values for spirometry for the 3–95-yr age range: the global lung function 2012 equations. European Respiratory Journal 2012;40: 1324–43.

[E5] Heaney LG, Busby J, Hanratty CE et al. Composite type-2 biomarker strategy versus a symptom–risk-based algorithm to adjust corticosteroid dose in patients with severe asthma: a multicentre, single-blind, parallel group, randomised controlled trial. Lancet Respir Med. 2021 Jan; 9 (1): 57–68.

[E6] Rosenkranz B, Kitajima W, Frölich JC. Relevance of urinary 6-keto-prostaglandin F1α determination. Kidney Int. 1981 Jun; 19 (6):755–9.

[E7] Schlondorff D. Renal prostaglandin synthesis: Sites of production and specific actions of prostaglandins. Am J Med. 1986 Aug; 81 (2): 1–11.
